# Multiple Lines of Evidence for Independent Origin of Wild and Cultivated Flowering Cherry (*Prunus yedoensis*)

**DOI:** 10.3389/fpls.2019.01555

**Published:** 2019-12-19

**Authors:** Myong-Suk Cho, Seung-Chul Kim

**Affiliations:** Department of Biological Sciences, Sungkyunkwan University, Suwon, South Korea

**Keywords:** chlorotype network, hybrid origin, Jeju Island, *Prunus yedoensis*, reticulation, Somei-yoshino

## Abstract

As with many other ornamental and cultivated plants that have been under human selection and cultivation for a long time, it has been a major challenge to trace back the complex evolutionary history of flowering cherry, *Prunus yedoensis*. This challenge has been further amplified by great morphological similarities, little molecular divergence, frequent natural and artificial hybridization, and poor documentation of breeding history among cultivated and wild flowering cherries. The origin and taxonomic distinction between wild *P. yedoensis* from Jeju Island, Korea, and one of the most popular cultivated flowering cherries, *P.* × *yedoensis* “Somei-yoshino” has been a controversy for the past few decades. We sampled many areas extensively, and using four different molecular markers we provided evidence for their independent origin. Wild *P. yedoensis* in Korea originated from multiple bidirectional hybridization events between two sympatric species, *P. spachiana* f. *ascendens* as the maternal species and *P. serrulata* var. *spontanea*/*P. serrulata* var. *quelpaertensis* as the most probable paternal species. On the contrary, our results supported a single artificial hybrid origin of *P.* × *yedoensis* “Somei-yoshino” from cultivated *P. spachiana* f. *ascendens* as the maternal species and *P. speciosa*, a species endemic to Izu Islands, as the paternal species. Based on extensive sampling, we provided strong evidence that wild and cultivated *P. yedoensis* are distinct taxonomic entities that have originated from different evolutionary processes. A potential for the development of new cultivars from wild *P. yedoensis* and conservation of diverse germplasms *in situ* insular setting and *ex situ* should be explored in the future.

## Introduction

The genus *Prunus* L., belonging to the family Rosaceae, subfamily Amygdaloideae, includes approximately 250 species of shrubs and trees, which are most abundantly distributed in the temperate zone of the Northern Hemisphere, and about 75 species are distributed in the tropical and subtropical regions and in the Andes (Rehder, 1940; Kalkmann, 1965). Because of its large size and wide distribution, the genus *Prunus* includes many economically important species of edible fruit as well as ornamental, medicinal, and timber species (Ingram, 1948; Elias, 1980; Zomlefer, 1994). From a wide variety of ornamental and cultivated plants, flowering cherries (*Prunus* subgenus *Cerasus*) are undoubtedly the most popular trees in public and residential landscapes (gardens, parks, and streets). Many forms of ornamental flowering cherries with diverse origins and traits are being cultivated and a wide range of wild flowering cherry species are found in the forests of eastern Asia. The distinction between wild and cultivated flowering cherries is often somewhat ambiguous, as they share several very similar features that make it challenging to discriminate them. Additionally, cross-compatibility, especially among closely related species, has allowed for interspecific hybridization with more than one naturally occurring species to contribute to genetic and morphological diversity of some cultivars (Potter, 2011). Among hundreds of forms of flowering cherries, *P.* × *yedoensis* “Somei-yoshino” is by far the most common and widespread in East Asia (Korea and Japan) and in the United States (Fujino, 1900; Bailey and Bailey, 1976; Cheng et al., 2000). Wild *Prunus yedoensis*, which was first collected by Taquet in 1908 and reported by Koehne in 1912, occurs naturally on Jeju Island, Korea, and it has very similar morphological features as the cultivated *P.* × *yedoensis* “Somei-yoshino.” As a result of nearly indistinguishable morphological characteristics of wild *P. yedoensis* and cultivated *P.* × *yedoensis* as well as the unsolved question of the ambiguous origin of cultivated *P.* × *yedoensis*, their taxonomic identities and origins have been debated for over several decades.

**Figure 1:**
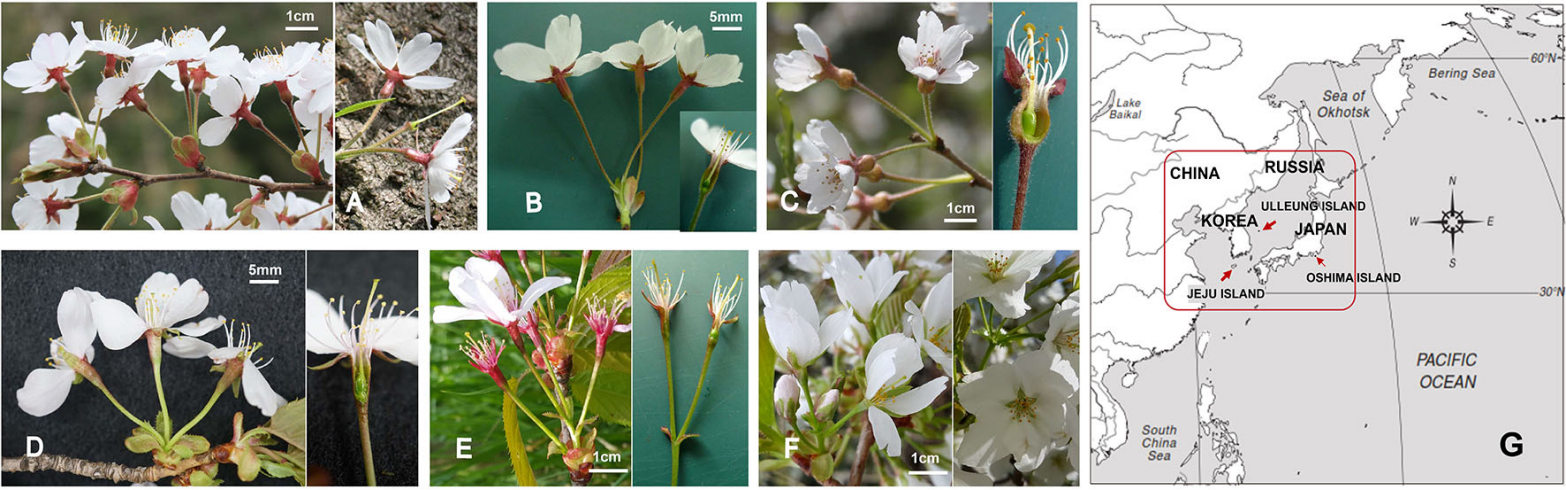
Variation in floral morphology of subg. *Cerasus* in wild *Prunus yedoensis* **(A)**, cultivated *P.* × *yedoensis* **(B)**, and their putative parental species, i.e., the putative maternal parent species, *P. spachiana* f. *ascendens* **(C)**, and the candidate paternal parent species, *P. sargentii* **(D)**, *P. serrulata* var. *spontanea* **(E)**, and *P. speciosa* **(F)**. The sampling sites are marked with red box on the map **(G)**.

The species *Prunus yedoensis* was originally described based on the cultivated hybrid of an unknown origin (Matsumura, 1901) without known wild populations in Japan (Krüssmann, 1986). The first record of planted *Prunus yedoensis* was in 1884 in Ueno Park, Tokyo (Kuitert, 1999); however, it is presumed that it has probably been planted before, possibly in the 18^th^ or early 19^th^ century (Iketani et al., 2007). It was propagated abundantly and it soon spread throughout Japan and other countries in Asia, Europe, and the United States. The lack of information on its origin has initiated investigations of various types of morphological traits (Wilson, 1916), crossing experiments (Takenaka, 1963), bibliographic studies (Iwasaki, 1991), chloroplast DNA (cpDNA) restriction fragment length polymorphisms (RFLP) analysis (Kaneko et al., 1986), DNA fingerprinting analysis (Innan et al., 1995), molecular phylogenetic studies (Ohta et al., 2006; Nakamura et al., 2015), and population genetic studies (Kato et al., 2014). Although it has not been definitely determined yet, cultivated *P.* × *yedoensis* is presumed to be a hybrid between *P. spachiana* f. *ascendens* (Makino) Kitam. [= *P. subhirtella* var. *ascendens* (Makino) E.H. Wilson = *P. pendula* Maxim.] and *P. speciosa* (Koidz.) Ingram [= *P. lannesiana* (Carr.) Wils. var. *speciosa* (Koidz.) Makino = *P. serrulata* Lindl. var. *lannesiana* Auct.] (**Figure 1**). Wilson (1916) was the first to propose this hybrid origin of *P.* × *yedoensis*, and his conclusion was supported by Takenaka's (1963) reciprocal crossing experiments between the parent species. *P. speciosa* was once suggested to be the maternal contributor (Funazu, 1966), but after analyses that included RFLP (Kaneko et al., 1986), DNA fingerprinting (Innan et al., 1995), and cpDNA *rpl*16-*rpl*14 spacer (Ohta et al., 2006) and nuclear *PolA1* gene sequencing (Nakamura et al., 2015), *P. spachiana* f. *ascendens* was proposed to be the female parent and *P. speciosa*, the male parent. In particular, DNA fingerprinting (Innan et al., 1995) and microsatellite studies (Iketani et al., 2007) confirmed its clonal origin from a single tree that was vegetatively propagated and spread all over Japan and beyond. The question of the origin of this cultivar may be difficult or even impossible to answer empirically, since no natural populations of *P.* × *yedoensis* have yet been found in Japan (Krüssmann, 1986; Iketani et al., 2007). Takenaka's hypothesis that *P.* × *yedoensis* originated from the Izu Peninsula was rejected after exhaustive research on wild Somei-yoshino and *P. spachiana* f. *ascendens* in the areas of Izu Peninsula, Miura Peninsula, and Boso Peninsula, where *P. speciosa*, the putative paternal contributor of *P.* × *yedoensis*, is endemic (Iwasaki, 1991). Instead, a hypothesis of an artificial hybrid origin by an unknown gardener from Somei village, which was the center of ornamental horticulture in 19^th^ century Edo/Tokyo Metropolis, has been considered more plausible (Fortune, 1863; Iwasaki, 1991).

In contrast to the lack of natural stands of the cultivated *P.* × *yedoensis* in Japan, there are several populations of wild *P. yedoensis* native to Jeju Island (Park et al., 1984; Kim, 1998; Kim et al., 1998; Jung and Oh, 2005; Cho et al., 2014). Its origin has been explored in morphological, histological, palynological, molecular phylogenetic, and population genetic studies (Park, 1965; Harn et al., 1977; Park et al., 1984; Jung et al., 1997; Jung et al., 1998; Kim et al., 1998; Jung and Oh, 2005; Roh et al., 2007; Cho et al., 2014; Cho et al., 2017). To date, however, no definite conclusions have been drawn, and more importantly, the phylogenetic relationship between wild *P. yedoensis* and cultivated *P.* × *yedoensis* is still unresolved. In our earlier study (Cho et al., 2014), we obtained molecular and morphological evidence to support the theory that natural hybridization between two sympatric *Prunus* lineages from Jeju Island played an important role in the origin of wild *P. yedoensis*. Since chloroplasts are inherited maternally (Kaneko et al., 1986; Matsumoto et al., 1997; Brettin et al., 2000; Raspé, 2001), *P. spachiana* f. *ascendens* was identified as the maternal parent based on the results of chloroplast phylogeny analyses, but the paternal parent from the *P. serrulata*/*P. sargentii* complex was not specified. Since the species in the *P. serrulata*/*P. sargentii* complex were closely related, species discrimination within this group was not possible, resulting in difficulties in the precise determination of the paternal parent of wild *P. yedoensis*. The same study (Cho et al., 2014) also documented that natural hybridization associated with the origin of wild *P. yedoensis* perhaps took place more than once, suggesting a multiple bidirectional hybrid origin. In the cpDNA phylogeny, *P. yedoensis* was not monophyletic and it was revealed that individuals of *P. spachiana* f. *ascendens* with different cpDNA haplotypes contributed to different *P. yedoensis* as the maternal parent. The independent study corroborated these findings in our recent study where they determined the paternal contributor (*P. jamasakura* = *P. serrrulata*) and analyzed the frequency of F_1_ hybrids and backcrossing generations in wild *P. yedoensis* (Cho et al., 2017). However, they did not specifically address the taxonomic distinction between wild *P. yedoensis* and cultivated *P.* × *yedoensis*.

Even though both wild *P. yedoensis* and cultivated *P.* × *yedoensis* share the same maternal parent species, their paternal parents are most likely different species because *P. speciosa*, the putative paternal species of *P.* × *yedoensis*, is not native to Jeju Island. Therefore, if we wish to precisely determine the phylogenetic relationship between them and solve the highly controversial question of whether wild *P. yedoensis* and cultivated *P.* × *yedoensis* are of independent origin, it is crucial to test the hypothesis that they are distinct taxonomic entities by investigating contributions from different paternal species. As for the maternal species, even if *P. spachiana* f. *ascendens* is the maternal contributor, it is necessary to determine its role as the contributor in the origin of wild *P. yedoensis* and cultivated *P.* × *yedoensis* populations. These hypotheses, however, have never been rigorously tested with a broad and suitable sampling method that would include all paternal candidate species involved in the origin of wild and cultivated flowering cherries. Thus, in this study, we extensively sampled wild populations of *P. speciosa* in their natural habitats of Oshima Island, Kouzu Island, and Izu Peninsula in Japan. We hypothesized that the paternal species differed in wild *P. yedoensis* and cultivated *P.* × *yedoensis*. The paternal candidate species of wild *P. yedoensis* collected from Jeju Island were compared to *P. speciosa* using several informative nuclear DNA markers. In addition to this analysis, we attempted to evaluate the genetic variations between the maternal species using maternally inherited chloroplast markers with the goal of resolving the differences between wild and cultivated taxa based on a large sample of *P. spachiana* f. *ascendens* from Jeju Island in Korea and from Japan. According to the results of our earlier analysis of the phylogenetic relationships between wild *P. yedoensis* and cultivated *P.* × *yedoensis* by comparison of chloroplast DNA sequences, some haplotypes of *P. spachiana* f. *ascendens* occurred exclusively in one region (i.e., Japan and Jeju Island, Korea), while others occurred in two regions (Cho, M.-S., unpublished data). Therefore, the aim of this study was to determine the phylogenetic relationship between wild *P. yedoensis* and cultivated *P.* × *yedoensis* based on biparently inherited nuclear markers and maternally inherited chloroplast makers. Specifically, we hoped to address the following questions: (1) Is there concrete evidence to support independent hybrid origin of wild *P. yedoensis* and cultivated *P.* × *yedoensis*?, (2) What are the parental species that contributed to the hybrid origin of wild *P. yedoensis* on Jeju Island?, and (3) What is the origin of cultivated *P.* × *yedoensis* “Somei-yoshino”? By addressing these questions, we aimed to elucidate the spontaneous hybridization process and artificial selection that are parts of the enigmatic origin of the cultivated flowering cherry. We also hoped that our extensive sampling and use of diverse molecular markers would solve the debates about the taxonomic entities of wild and cultivated flowering cherries that have been ongoing for several decades.

## Materials and Methods

### Plant Material

We sampled a total of 263 individuals belonging to 18 species and one cultivar (*P.* × *yedoensis* “Somei-yoshino”) from Jeju Island, Korean peninsula, Russia, and Japan (**Table S1**). For the ingroup, we extensively sampled the following 13 taxa of our main interest belonging to subgenus *Cerasus*, section *Pseudocerasus*; wild *P. yedoensis* on Jeju Island in Korea (29 individuals), *P. spachiana* f. *ascendens* (65 individuals; 26 from Korea and 39 from Japan), *P. sargentii* Rehder (40 individuals; 27 from Korea, 5 from Russia, and 8 from Japan), *P. serrulata* var. *spontanea* (Max) Wilson (12 individuals; 7 from Korea and 5 from Japan), *P. serrulata* var. *quelpaertensis* (Nakai) Uyeki (10 individuals from Jeju Island), *P. serrulata* var. *pubescens* (Makino) Nakai (4 individuals; 3 from Korea and 1 from Japan), *P. yedoensis* var. *angustipetala* Kim & Kim (1 individual from Jeju Island), *P. hallasanensis* Kim & Kim (2 individuals from Jeju Island), *P. longistylus* Kim & Kim (1 individual from Jeju Island), *P. speciosa* (Koidz.) Ingram (40 individuals; 3 from Korea and 37 from Japan), *P*. *takesimensis* Nakai (35 individuals from Ulleung Island, Korea), *P*. *incisa* Thunb. (1 individual from Japan), and *P*. *apetala* (Siebold & Zucc.) Franch & Sav. (3 individuals from Japan). In addition, we sampled *P. maximowiczii* Ruprecht (three individuals from Korea) from the section *Phyllomahaleb* and *P*. *avium* (L.) L. (one individual from Japan) from the section *Eurocerasus*. Three taxa, *P. longisylus*, *P. hallasanensis*, and *P. yedoensis* var. *augustipetala*, are very rare taxa endemic to Jeju Island, which is why only a few individuals of these species were sampled. Of 40 P*. speciosa* individuals, three individuals sampled from Jeju Island were cultivated plants, while the remaining 37 individuals from Japan included eight cultivated and 29 wild plants (of which 14 from Oshima Island, 8 from Kozushima Island, and 7 from Izu Peninsula). Therefore, this study was the first one to include all possible parental species involved in the origin of wild *P. yedoensis* and cultivated *P.* × *yedoensis*. We included five individuals from Koishikawa Botanical Garden, Tokyo, Japan, and seven individuals from Korea (two from Jeju National University, two from Seoul National University, and three from Jinhae Naval Base) in the samples of cultivated *P.* × *yedoensis*. The samples collected from Koishikawa Botanical Garden were planted in the 19^th^ century and represent a single clone type of cultivated *P.* × *yedoensis* “Somei-yoshino” that is spread throughout Japan (Iketani et al., 2007). For outgroup taxa, we sampled three species of *Prunus* subgenus *Padus*: *P. padus* L. (one individual from Korea), *P. grayana* Maxim. (one individual from Japan), and *P. buergeriana* Miq. (one individual from Korea and one from Japan).

Of a total of 263 individuals, 194 individuals that included all the required species were used for phylogenetic analyses based on nuclear ribosomal DNA internal transcribed spacer region (nrDNA ITS) and external transcribed spacer region (ETS) as well as seven combined cpDNA noncoding regions. The analyses of paternal species of wild *P. yedoensis* and cultivated *P.* × *yedoensis* were conducted using 87 individuals for Rosaceae Conserved Orthologous Set (RosCOS) data set and 42 individuals for single copy nuclear *PolA1* gene data set (Nakamura et al., 2015) (**Table S1**). For the analysis of the cpDNA haplotype network, 105 individuals of wild *P. yedoensis*, *P.* × *yedoensis*, and *P. spachiana* f. *ascendens* were used. All voucher specimens were deposited at the Ha Eun Herbarium, Sungkyunkwan University (SKK), Korea.

### DNA Isolation, Molecular Markers, PCR Amplification, Sequencing, and Sequence Alignment

Silica-gel dried leaves collected mostly from natural populations with several accessions from botanical gardens were used as DNA sources, and DNA extraction was done using the DNeasy Plant Mini Kit (Qiagen, Carlsbad, California, USA). Nuclear ITS and ETS DNA regions and seven highly variable noncoding regions of chloroplast DNA (*pet*A-*psb*J, *pet*D-*rpo*A, *ndh*F-*rpl*32, *trn*Q-*rps*16, *trn*V-*ndh*C, *rpl*16 intron, and *trn*L-*rpl*32; Shaw et al., 2007) were amplified for phylogenetic analyses. To corroborate and gain additional insights into their phylogenetic relationships, we sequenced the single copy nuclear *PolA1* gene that encodes nuclear RNA polymerase A1 as well as single copy Rosaceae Conserved Orthologous Set (RosCOS) markers. *PolA1* containing intron PI19 and exon PE20 sequence was amplified using forward primer 19ex5P and reverse primer 21ex3P (Nakamura et al., 2015) (**Table S2**). Several accessions of proposed hybrids, wild, and cultivated *P. yedoensis* were cloned to obtain clean sequences and distinguish maternal and paternal contribution in the intron PI19 region. PI19 intron data set included 25 accessions of cloned sequences of wild *P. yedoensis* (5191000 and 868), cultivated *P.* × *yedoensis* (KO4972 and KO5033), and *P. serrulata* var. *quelpaertensis* (QP838) (**Table S1**). Additional internal primers 20ex5P and 20ex3P were also used for amplification and sequencing.

RosCOS are highly effective genetic markers that are mostly single-copy and evolutionarily conserved among the members of Rosaceae family. A total of 26 RosCOS markers evenly spaced across eight chromosomes were randomly chosen (Genome Database for *Rosaceae* ^1^) (**Figure S1**). Among the 26 tested RosCOS markers (**Table S3**), four markers (RosCOS_00517, 03628, 01167, and 01445) were capable of distinguishing between different polymorphisms in the putative paternal contributors of wild *P. yedoensis* and cultivated *P.* × *yedoensis*. Therefore, these four RosCOS markers were amplified in 87 accessions of wild and cultivated *P. yedoensis* and their putative parental species in order to determine their genetic contribution to wild and cultivated flowering cherries. For the analysis of chloroplast DNA haplotype network and determining the maternal contribution species, five highly informative regions (*pet*B-*pet*D, *pet*D-*rpo*A, *trn*S-*trn*G, *rpl*16 gene, and *ycf*1 gene) were selected based on the comparison of whole chloroplast genomes of wild *P. yedoensis* and cultivated *P.* × *yedoensis* (Cho et al., 2018). All primer pairs used for amplification are specified in **Table S2**. The thermal cycler program was run as follows: one cycle of 95°C for 2 min (initial denaturation), 35 cycles of 20 s at 95°C (denaturation), 40 s at 52°C (annealing), and 1 min at 72°C (extension), and finally 5 min of 72°C (final extension). All PCR products were purified with the Inclone^™^ Gel & PCR Purification Kit (InClone Biotech Co., Seoul, Korea). Direct sequencing of the purified PCR products was carried out using BigDye Terminator v3.1 Cycle Sequencing Kit (Applied Biosystems, Foster City, California) at the Geno Tech Corp. (Daejeon, Korea). Contig assembly and editing were performed using Geneious ver. 8.1.7 (Kearse et al., 2012). In order to determine additive polymorphic sites (APS) using direct sequencing of ITS and ETS sequences, cloning of the ITS regions obtained from the PCR was conducted as described in Cho et al. (2014). We cloned the ITS regions of several representative species, including wild *P. yedoensis* (four samples, 826_1, 826_2, 868, and 880), cultivated *P.* × *yedoensis* (five samples, SNU_K, KO3492, KO4972, KO4981, and KO5041), *P*. *spachiana* f. *ascendens* (two samples, 820 and 857), *P. serrulata* var. *quelpaertensis* (one sample, 838), *P. serrulata* var. *spontanea* (one sample, 816), *P. sargentii* (one sample, 861) and *P. speciosa* (two samples, KO89_00277 and KO89_251).

### Phylogenetic Analysis

We conducted maximum parsimony (MP) and maximum likelihood (ML) analyses of the nuclear ITS/ETS data set (194 individuals and 144 accessions of cloned ITS amplicons) and of the chloroplast data set (194 individuals and seven noncoding regions). The Fitch parsimony (Fitch, 1971) implemented in PAUP ver. 4.0 (Swofford, 2002) was used for MP analysis with default heuristic search options; starting trees *via* stepwise addition, simple sequence addition with one tree held at each step, tree-bisection-reconnection (TBR) branch swapping, steepest descent and MulTrees options on, zero branch length collapsed, and topological constraints not enforced. Bootstrap support (BS) for the robustness of groups was calculated from 1,000 bootstrap replicates (Felsenstein, 1985) with the same heuristic settings. The polymorphic sites of ITS and ETS were treated as polymorphisms. ML analysis was conducted with RaxmlGUI ver. 1.5 (Silvestro and Michalak, 2012). The GTR+gamma model was chosen as the best-fit model of molecular evolution using RAxML rapid bootstrapping with 1,000 replicates. In order to determine the phylogenetic relationship of the maternal parent lineages between wild *P. yedoensis* and cultivated *P.* × *yedoensis*, a cpDNA haplotype network was constructed using statistical parsimony implemented in the program ARLEQUIN ver. 3.1 (Excoffier et al., 2005) from 105 accessions of wild *P. yedoensis*, cultivated *P.* × *yedoensis*, and *P. spachiana* f. *ascendens* collected from Jeju Island and Japan. Gaps were treated as missing data, and the connection limit (excluding homoplastic changes) was set to 95% in accordance with Hart and Sunday (2007). Paternity analysis was done by comparison of SNPs in the sequences of *PolA1* gene and in Rosaceae Conserved Orthologous Set (RosCOS) regions. Four RosCOS loci obtained by direct sequencing were aligned and their SNPs were determined using Geneious ver. 8.1.7. In addition, amplified DNA fragments of *PolA1* yielded the PI19 intron data set (41 accessions and 25 cloned accessions) and PE20 exon data set (42 accessions) from which we could determine SNPs for comparison between the putative paternal species. Four RosCOS markers (RosCOS_00517, 03628, 01167, and 01445) from a total of 87 accessions were visualized with a split graph obtained from SplitsTree ver. 4.14.2 using an equal angle algorithm (Huson and Bryant, 2006). Split networks were constructed using the neighbor-net method generated from inferred distance matrices. The edges were labeled with bootstrap probabilities of more than 50% calculated with a bootstrap analysis with 1,000 bootstrap replicates.

## Results

### Chloroplast and Nuclear DNA Phylogeny

**Figure 2:**
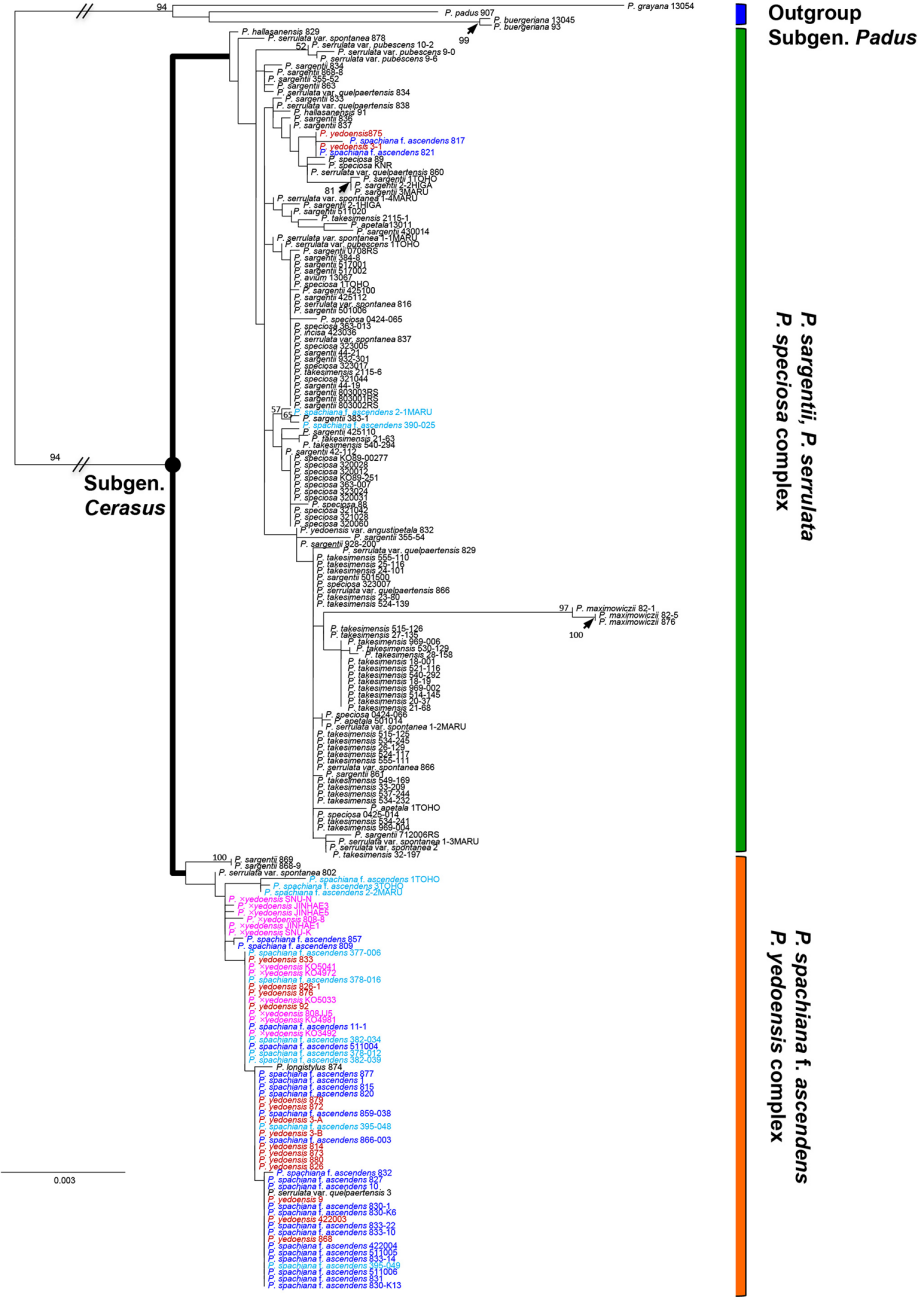
RAxML tree based on 194 accessions and seven concatenated cpDNA regions. Different colors represent different localities of the species in the *P. spachiana* f. *ascendens* and *P. yedoensis* complex: red for wild *P. yedoensis* from Jeju Island, pink for cultivated *P.* × *yedoensis* from Japan, royal blue for *P. spachiana* f. *ascendens* from Korea, and aqua blue for *P. spachiana* f. *ascendens* from Japan. Numbers above branches indicate bootstrap support (BS) percentages of >50% based on ML analysis.

As shown in our previous study (Cho et al., 2014), in the results of both nuclear and chloroplast phylogenies we only found a few well-supported clades. Nevertheless, regarding the relationship of wild *P. yedoensis* and cultivated *P.* × *yedoensis*, we can emphasize a few important findings of this research. In the results of chloroplast DNA phylogeny based on seven concatenated regions (194 accessions and 5,547 total characters; see data matrices available in dryad under doi: 10.5061/dryad.cz8w9gj02), two major lineages could be identified (**Figure 2**). The first lineage of *P. serrulata*/*P. sargentii*/*P*. *speciosa* complex included *P. maximowiczii* from the section *Phyllomahaleb, P*. *avium* from the section *Eurocerasus*, and *P*. *serrulata*, *P*. *sargentii*, *P*. *speciosa*, *P*. *takesimensis*, *P*. *apetala*, *P*. *incisa*, and other closely related species from the section *Pseudocerasus*. Several exceptional accessions of wild *P. yedoensis* (3-1 and 875) and *P. spachiana* f. *ascendens* (817, 821, 390_025, and 2_1) also belonged to this lineage. The second lineage of *P. spachiana* f. *ascendens*/*P*. *yedoensis* complex included *P. spachiana* f. *ascendens*, the majority of wild *P. yedoensis*, and all cultivated *P.* × *yedoensis* accessions, along with a few *P. sargentii* (868-9 and 869), *P. serrulata* var. *spontanea* (802), and *P. serrulata* var. *quelpaertensis* (3) accessions, which would otherwise be placed in the first lineage. The phylogeny of the maternally inherited cpDNA showed that both wild *P. yedoensis* and all cultivated *P.* × *yedoensis* share the most recent common ancestor with *P. spachiana* f. *ascendens*, corroborating the results of our previous study (Cho et al., 2014).

**Figure 3:**
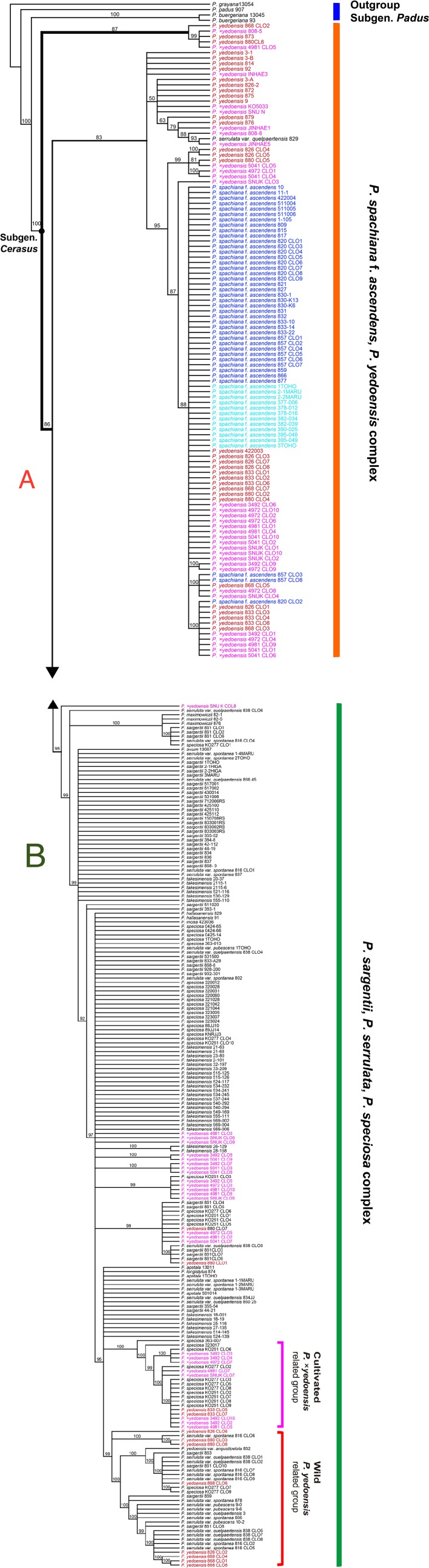
50% majority-rule consensus tree of 50,000 equally parsimonious trees based on nrDNA ITS regions, including 322 accessions of representative *Prunus* species. **(A)** Upper half of the phylogenetic tree. **(B)** Lower half of the phylogenetic tree. Different colors represent different geographical regions: red for wild *P. yedoensis* from Jeju Island, pink for cultivated *P.* × *yedoensis* from Japan; royal blue for *P. spachiana* f. *ascendens* from Korea, and aqua blue for *P. spachiana* f. *ascendens* from Japan. Numbers above branches indicate bootstrap support (BS) percentages of >50%.

The results of the ITS/ETS phylogeny based on direct sequencing (194 accessions and 956 total characters; not shown) revealed noticeable additive polymorphic sites (APS) in wild *P. yedoensis* and cultivated *P.* × *yedoensis* in the parental species-specific sites of two parental species, *P. spachiana* f. *ascendens* and *P. serrulata/P. sargentii/P. speciosa* complex (**Table S4**). These findings were highlighted in our previous study as well (Cho et al., 2014). We cloned the ITS regions of four wild *P. yedoensis* individuals (826-1, 833-2, 868, and 880) and five cultivated *P.* × *yedoensis* individuals (SNU-K, KO3492, KO4972, KO4981, and KO5041) to confirm the results of the direct sequencing. We also cloned two parental lineages of *P. spachiana* f. *ascendens* (820 and 857) and *P. serrulata*/*P. sargentii*/*P*. *speciosa* complex (861, 816, 838, KO89-00277, and KO89-251). The ITS tree based on 322 accessions (178 direct and 144 cloned sequences; see data matrices available in dryad under doi: 10.5061/dryad.cz8w9gj02) provided new insights into the origin of wild and cultivated flowering cherries. The first lineage in the ITS tree included directly sequenced accessions and all other cloned sequences of *P. spachiana* f. *ascendens*, the majority of directly sequenced accessions and some cloned ribotypes of wild *P. yedoensis*, and all cultivated *P.* × *yedoensis* (**Figure 3A**). The second lineage included all putative paternal parental species and all cloned ribotypes from their representative accessions of *P. serrulata* var. *spontanea* (816), *P. serrulata* var. *quelpaertensis* (838), *P. sargentii* (861), and *P*. *speciosa* (KO89-00277 and KO89-251) (**Figure 3B**), as well as other remaining cloned ribotypes of wild *P. yedoensis* and cultivated *P.* × *yedoensis*. Within the second lineage that consisted of putative paternal species, some cloned accessions of wild *P. yedoensis* and cultivated *P.* × *yedoensis* did not form distinguished monophyletic groups and they did not show any genetic closeness to *P*. *speciosa* or to the species distributed on Jeju Island. However, some clones were grouped separately into the wild *P. yedoensis* related group and the cultivated *P.* ×*yedoensis* related group. For example, seven cloned accessions of cultivated *P.* × *yedoensis* formed a distinct group that included 11 clones of *P*. *speciosa* and two cloned accessions of wild *P. yedoensis* (833-2) (**Figure 3B**). Another distinct group included eight cloned accessions of wild *P. yedoensis* (826, 868, and 880 accessions) as well as the directly sequenced and cloned accessions of the species from Jeju Island (excluding two cloned ribotypes of *P*. *speciosa*, KO89-00277 accession).

### Nuclear RosCOS Polymorphisms and Split Graph

**Figure 4:**
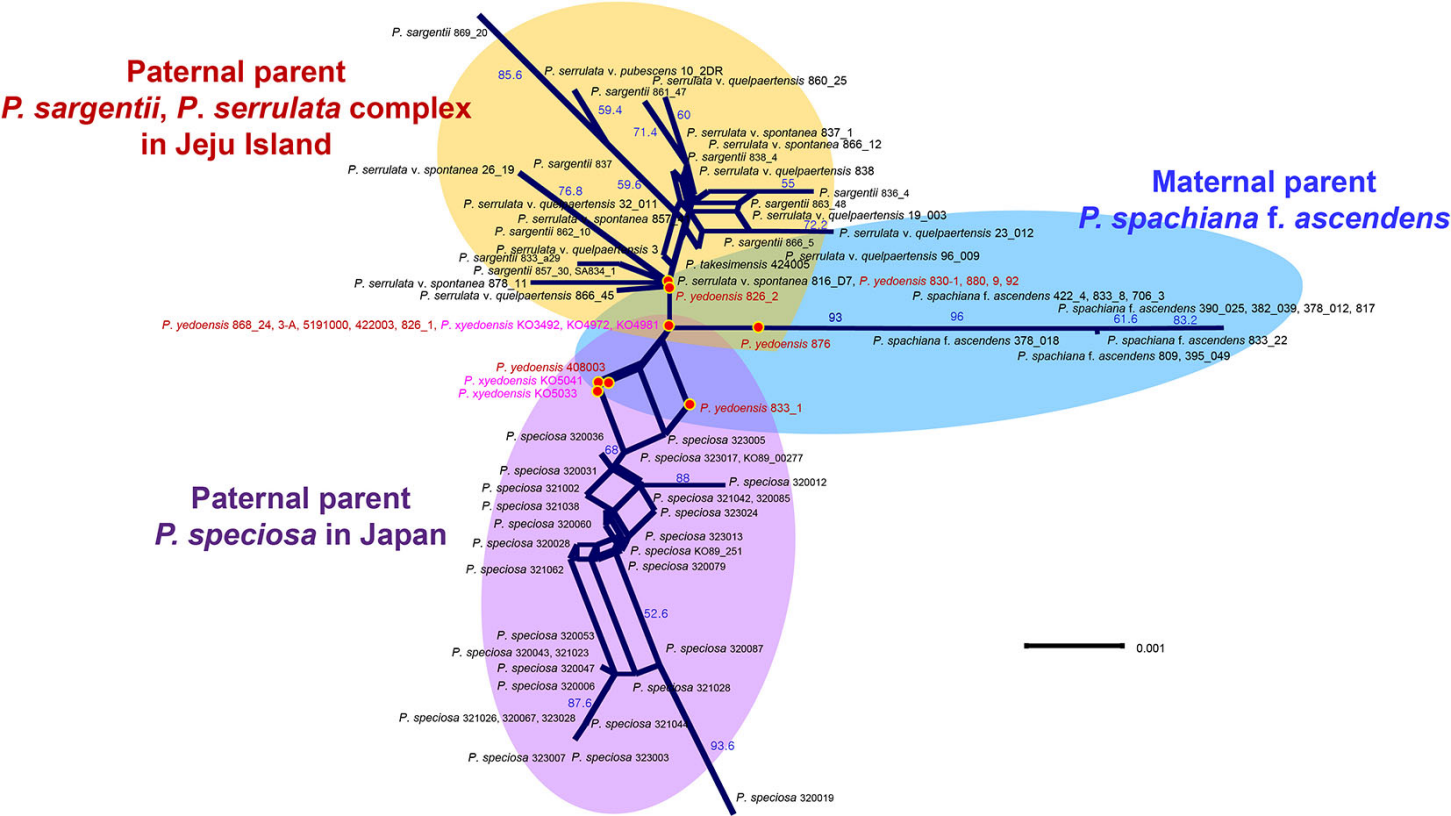
A neighbor-net split graph obtained based on four concatenated RosCOS markers for 89 accessions. Numbers above branches indicate bootstrap support (BS) percentages of >50%.

A total of 26 RosCOS loci (with a total of 11,730 characters) were screened for variation in eight representative species (*P. spachianana* f. *ascendens* 833_22, wild *P. yedoensis* 868, *P. sargentii* 866_5/861, *P. serrulata* var. *spontaneae* 816_1_D7, *P. serrulata* var. *quelpartensis* 838, *P. serrulata* var. *pubescens* 10-2, *P. speciosa* 323007, and *P. takesimensis* 969_005). The results of the nrDNA ITS/ETS analysis of 194 individuals showed a total of 58 APS in two parental species-specific sites (data not shown). Four of the 26 screened loci (RosCOS_00517, 03628, 01167, and 0 1445) were used to distinguish between wild *P. yedoensis* and cultivated *P.* × *yedoensis*. The results of the analysis of four RosCOS data sets (87 accessions and 1250 characters; see data matrices available in dryad under doi: 10.5061/dryad.cz8w9gj02) revealed 22 informative polymorphisms. In the sequences of wild *P. yedoensis* and cultivated *P.* × *yedoensis*, seven sites (188, 248, 278, 893, 934, 1047, and 1112) displayed APS, which were most likely contributions from two parental lineages. In the remaining 15 sites, wild *P. yedoensis* and cultivated *P.* × *yedoensis* differed from each other by having different nucleotides transferred from their paternal parents, and their maternal parent contributions had the same sequences in the same researched sites (**Table S5**). A split graph drawn for four RosCOS data sets was composed of 62 splits with a neighbor-net network of 95 vertices and 126 edges (**Figure 4**). Hybridization hypothesis was visualized on the network and three major groups, *P. spachiana* f. *ascendens*, *P. speciosa* from Japan, and *P. serrulata*/*P. sargentii* complex from Jeju Island, could be seen. The hybrid accessions of wild *P. yedoensis* and cultivated *P.* × *yedoensis* were placed in the center of the split graph without unique patterns (i.e., no edges). Wild *P. yedoensis* and cultivated *P.* × *yedoensis* constructed eight split networks that were generally separated from each other except for one network that included five wild and three cultivated accessions.

### *PolA1* Data

*PolA1* data sets for PI19 intron (41 accessions) and PE20 exon (42 accessions) included cultivated *P.* × *yedoensis* and its putative parents *P. spachiana* f. *ascendens* and *P. speciosa* collected from Japan as well as wild *P. yedoensis* and its putative parent species collected from Jeju Island, Korea. The 25 cloned accessions of wild *P. yedoensis* (5191000 and 868), cultivated *P.* × *yedoensis* (KO4972 and KO5033), and *P. serrulata* var. *quelpaertensis* (QP838) were also included in the PI19 intron data set. In addition, we obtained the sequences of cultivated *P*. × *yedoensis* and its putative parental species from the DNA Data Bank of Japan (Nakamura et al., 2015), and we combined this data with our data set (see data matrices available in dryad under doi: 10.5061/dryad.cz8w9gj02). The sequences obtained by Nakamura et al. (2015) were identical to the ones of cultivated *P. yedoensis* and *P. speciosa* obtained from our collection from Japan. The results of the analysis of the combined data set of PI19 intron region (a total of 72 accessions, including six downloaded ones, 25 cloned and 41 directly sequenced accessions, with a total of 630 characters; see data matrices available in dryad under doi: 10.5061/dryad.cz8w9gj02) showed 25 informative SNPs for discriminating hybrid accessions (**Table S6**). Specifically, seven informative sites (1, 61, 139, 209, 430, 492, and 495) that resulted from different maternal or paternal contributions enabled us to differentiate between wild *P. yedoensis* and cultivated *P.* × *yedoensis*. For example, based on four sites (1, 61, 139, and 209), cultivated *P.* × *yedoensis* belonged to two haplotypes, one of which was shared with *P. speciosa* that had different nucleotides from those of the paternal lineage of wild *P. yedoensis* on Jeju Island. As a result, wild *P. yedoensis* on Jeju Island had different nucleotides from cultivated *P.* × *yedoensis* as confirmed by the results of our analyses. Nakamura et al. (2015) suggested the that the maternal parent of cultivated *P.* × *yedoensis* could be a related cultivar “Komatsu-otome” (LC010415*_P. pendula*) instead of wild *P. spachiana* f. *ascendens* based on the sequence of site 430, since the “K” haplotype of cultivated *P.* × *yedoensis* was identical to “Komatsu-otome.” Neither wild *P. yedoensis* nor its maternal parent shared the “K” haplotype, suggesting different maternal lineages for wild *P. yedoensis* and cultivated *P.* × *yedoensis*. In case of sites 492 and 495, a small part of wild *P. yedoensis* accessions were found to have different nucleotides from cultivated *P.* × *yedoensis*, which was presumably a result of the variations (probably more than one) in paternal parent species. The combined data set of PE20 exon region (a total of 47 accessions, including five downloaded accessions and 42 directly sequenced accessions, with a total of 764 characters; see data matrices available in dryad under doi: 10.5061/dryad.cz8w9gj02) found eight SNPs, seven of which were informative (121, 463, 471, 534-557, 676, 705, and 712), and this allowed us to discriminate between the contributions of different parental species in wild *P. yedoensis* and cultivated *P.* × *yedoensis* (**Table S7**).

### Haplotype Network of cpDNA

**Figure 5:**
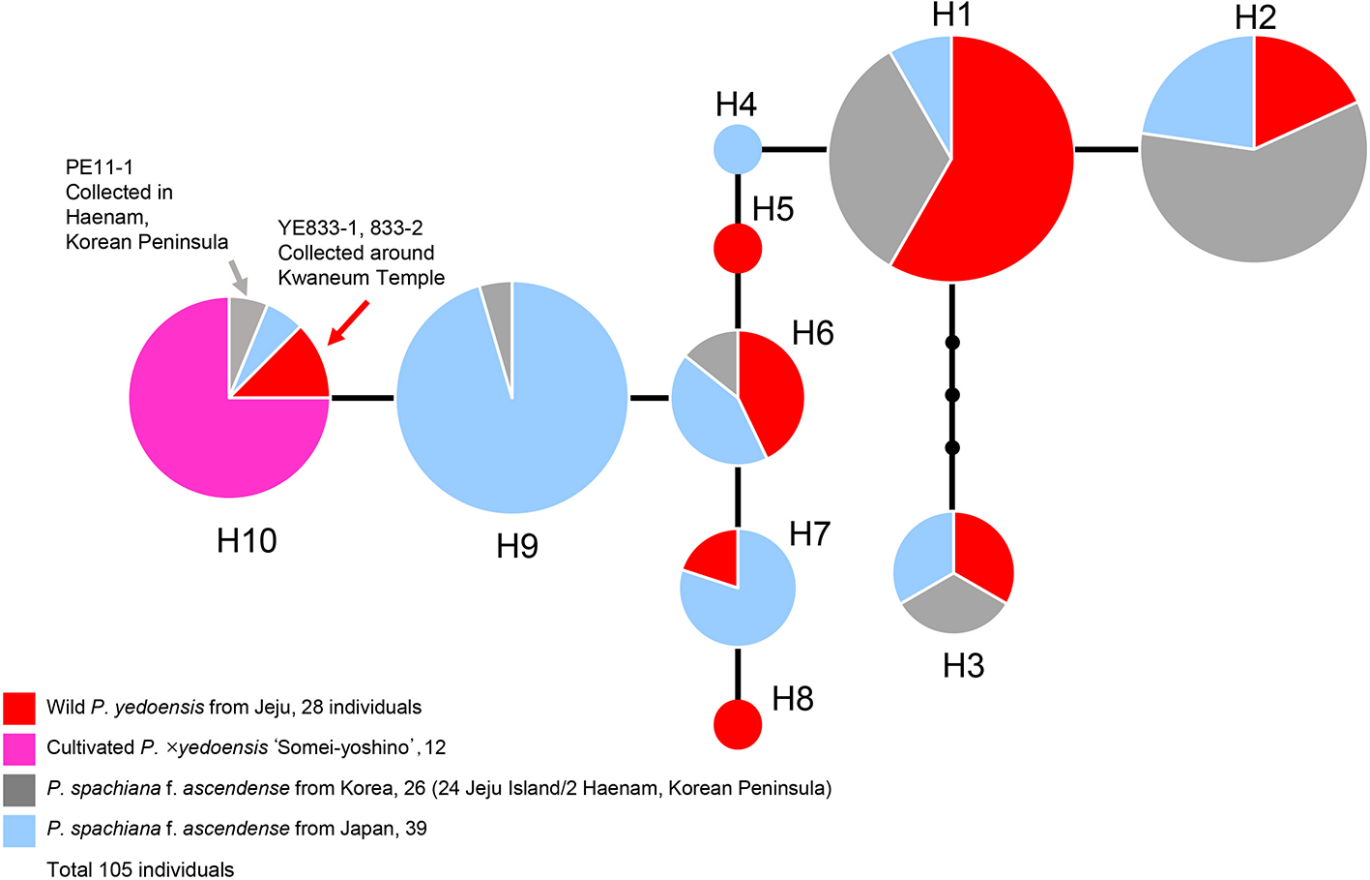
A haplotype network obtained based on five concatenated cpDNA regions of wild *P. yedoensis*, cultivated *P.* × *yedoensis*, and *P. spachiana* f. *ascendens* from Korea and Japan. Different colors within each haplotype are proportional to the frequency of each taxon in the haplotype. Sizes of circles are proportional to the number of individuals that is written in parentheses.

A concatenated data set (105 accessions and 2,826 characters; see data matrices available in dryad under doi: 10.5061/dryad.cz8w9gj02) of five chloroplast regions (*pet*B-*pet*D, *pet*D-*rpo*A, *trn*S-*trn*G, *rpl*16, and *ycf*1) was analyzed with the ARLEQUIN software in order to construct the haplotype network. Ten haplotypes were identified among 105 accessions of wild *P. yedoensis*, cultivated *P.* × *yedoensis*, and their common maternal parent *P. spachiana* f. *ascendens* collected from Jeju Island and Japan. The haplotype network illustrating their genealogical relationship is shown in **Figure 5** and the members of each haplotype are specified in **Table 1**. We found that the wild *P. yedoensis* had eight highly diverse haplotypes (H1, H2, H3, H5, H6, H7, H8, and H10) mostly shared with *P. spachiana* f. *ascendens*, while the cultivated *P.* × *yedoensis* only had one haplotype (H10). The most frequent H1 haplotype was shared between wild *P. yedoensis* (14 accessions) and *P. spachiana* f. *ascendens* collected from Jeju Island (8 accessions) and Japan (2 accessions). H2 haplotype (22 accessions) was closely related to H1 haplotype (differing by a single mutational step) and included the same combination of wild *P. yedoensis* and *P. spachiana* f. *ascendens* collected from Jeju Island and Japan. The H3 haplotype differed from H1 in three missing (or inferred) haplotypes. This somewhat divergent H3 haplotype was present in six individuals of wild *P. yedoensis* (two accessions), *P. spachiana* f. *ascendens* collected from Japan (two individuals), and *P. spachiana* f. *ascendens* collected from Korea (two individuals). H3 is a typical cpDNA haplotype of *P. serrulata*/*P. sargentii*/*P*. *speciosa* complex and it is a result of a reverse direction of hybridization: instead of *P. spachiana* f. *ascendens* being the maternal parent, species from this complex served as the maternal parent (Cho et al., 2014). Three very rare haplotypes were found, H4 in *P. spachiana* f. *ascendens* from Japan, and H5 and H8 in wild *P. yedoensis* from Jeju Island. As is the case with the common H1 and H2 haplotypes, H6 haplotype was shared by wild *P. yedoensis* (three accessions) and *P. spachiana* f. *ascendens* (one accession from Korea and three from Japan). The H7 haplotype was shared only by wild *P. yedoensis* (one accession) and *P. spachiana* f. *ascendens* from Japan (four accessions). Considering the independent origin of wild and cultivated forms, it is possible that we did not collect this particular haplotype from Jeju Island. The H9 haplotype was shared by 21 individuals of *P. spachiana* f. *ascendens* from Japan, but very rarely by species from Korea (one accession). Lastly, the H10 haplotype included all accessions of cultivated *P.* × *yedoensis* (12 accessions from Korea and Japan) and was shared by *P. spachiana* f. *ascendens* collected from Jeju Island and Japan (one accession from each country). Two additional accessions of wild *P. yedoensis* collected around Kwaneum Temple on Jeju Island (YE833-1 and 833-2) also belonged to this haplotype. These two individuals most likely represented individuals escaped from cultivation near the temple area (Cho et al., 2014). In summary, wild *P. yedoensis* shared cp haplotypes (H1, H2, H3, and H4) with its putative maternal parent *P. spachiana* f. *ascendens* collected from Jeju Island and Japan. In rare cases, we found *P. spachiana* f. *ascendens* haplotype (H4) exclusively in Japan and wild *P. yedoensis* haplotype (H5 and H8) in Korea. These two haplotypes found in wild *P. yedoensis* most likely represent undetected haplotypes of *P. spachiana* f. *ascendens* involved in the origin of wild *P. yedoensis*. No unique haplotype of *P. spachiana* f. *ascendens* was found in Korea. Given the fact that all cultivated *P.* × *yedoensis* shared the H10 haplotype and were separated from the remaining haplotypes, genetic distinction from wild and cultivated forms was possible.

**Table 1:** Chloroplast haplotypes of 105 accessions of wild *P. yedoensis*, cultivated *P.* × *yedoensis*, and their maternal parental species, *P. spachiana* f. *ascendens*.

Haplotype	Numbers of accessions	Description of accessions
**H1**	24	Wild *P. yedoensis* (14): 836-9, 867_19, 94_3, 3_A, 3_B, 5191000, 814, 816, 826_2, 857_34, 872, 873, 879, 880 *P. spachiana* f. *ascendens* from Jeju (8): 1, 815, 820, 833-8, 857, 859_038, 866_003, 877 *P. spachiana* f. *ascendens* from Japan (2): 706_3, 395_048
**H2**	22	Wild *P. yedoensis* (4): 830, 422003, 868, 9 *P. spachiana* f. *ascendens* from Jeju (13): 10 (Korean Peninsula), 422004, 511005, 511006, 827, 830_1, 830_K13, 830-K6, 821, 832, 833_10, 833_14, 833_22 *P. spachiana* f. *ascendens* from Japan (5): 706_4, 706_9, 395_049, 706_2, 706_7
**H3**	6	Wild *P. yedoensis* (2): 3_1, 875 *P. spachiana* f. *ascendens* from Jeju (2): 817, 821 *P. spachiana* f. *ascendens* from Japan (2): 2_1MARU, 390_025
**H4**	1	*P. spachiana* f. *ascendens* from Japan (1): 706_8
**H5**	1	Wild *P. yedoensis* (1): 836
**H6**	7	Wild *P. yedoensis* (3): 826_1, 876, 92 *P. spachiana* f. *ascendens* from Jeju (1): 809 *P. spachiana* f. *ascendens* from Japan (3): 378_018, 706_13, 378_016
**H7**	5	Wild *P. yedoensis* (1): 871 *P. spachiana* f. *ascendens* from Japan (4): 378_008, 517001, 1TOHO, 3TOHO
**H8**	1	Wild *P. yedoensis* (1): 804
**H9**	22	*P. spachiana* f. *ascendens* from Jeju (1): 511004 *P. spachiana* f. *ascendens* from Japan (21): 378_013, 927_102, 706_1, 706_5, 706_6, 706_11, 706_12, 706_14, 706_15, 706_17, 706_18, 706_19, 706_21, 706_10, 706_16, 706_20, 706_22, 2_2MARU, 377_006, 378_012, 382_034,
**H10**	16	Wild *P. yedoensis* (2): 833_1, 833_2 Cultivated *P.* × *yedoensis* (12): 808_5, 808_8, JINHAE1, JINHAE3, JINHAE5, KO3492, KO4972, KO4981, KO5033, KO5041, SNU_K, SNU_N *P. spachiana* f. *ascendens* from Korean Peninsula (1): 11-1 *P. spachiana* f. *ascendens* from Japan (1): 382-039

## Discussion

### Evolutionary Origin and Taxonomic Distinction Between Flowering Cherries

In this study, based on four lines of evidence from the analysis of three independent nuclear (nrDNA ITS, RosCOS SNPs, and single copy nuclear gene *PolA1*) and chloroplast loci, we documented that independent natural and artificial hybridization events have occurred in wild *P. yedoensis* and cultivated *P.* × *yedoensis*, respectively. We determined the parentages of the two taxonomic entities. In case of the paternal parent, it is most likely that the species *P. serrulata* var. *spontanea*/*P. serrulata* var. *quelpaertensis* sympatrically distributed on Jeju Island contributed to the origin of wild *P. yedoensis*. On the other hand, *P. speciosa*, which occurs naturally in the Izu Islands and Izu Peninsula, was determined as the paternal parent of cultivated *P.* × *yedoensis*. From the obtained maternally inherited cpDNA haplotype network we concluded that *P. spachiana* f. *ascendens*, which occurs on Jeju Island, southern part of the Korean peninsula, and the Japanese archipelago, contributed to the origin of both wild *P. yedoensis* and cultivated *P.* × *yedoensis* as the maternal parent. However, *P. spachiana* f. *ascendens* plants involved in the origin of two taxonomic entities had a different genetic background; wild *P. yedoensis* originated from multiple wild *P. spachiana* f. *ascendens* individuals from Jeju Island, whereas most likely a single cultivated plant of *P. spachiana* f. *ascendens* was involved in the origin of cultivated *P.* × *yedoensis* in Japan.

Although the potential hybrid origin of wild and cultivated flowering cherries can be traced back to the 20^th^ century, it was only in 2007 that Roh et al. proposed the hypothesis of an independent origin of wild *P. yedoensis* and cultivated *P.* × *yedoensis* with a possibility of these species being distinct taxonomic entities. Based on the chloroplast (*rpl*16 intron and *trn*L-F intergenic spacer) and ISSR markers, they suggested that wild *P. yedoensis* from Jeju Island differs sufficiently from the cultivated Somei-yoshino cherry from Japan. However, their conclusions were poorly supported; they overlooked the fact that the several accessions of Somei-yoshino cherries (1, 2, 122, and 123) showed a close relationship with *P. yedoensis* on the ISSRs phenogram, and that two haplotypes from cpDNA sequences of the *rpl*16 intron overlapped [the wild “TA” haplotype and the cultivated *P. yedoensis* (“AA” haplotype)]. Based on this phenogram and cpDNA haplotype patterns, it can be concluded that Somei-yoshino cherries originated from the wild *P. yedoensis* from Jeju Island and that wild *P. yedoensis* and cultivated *P.* × *yedoensis* are taxonomically identical, which is contrary to their conclusions. Nevertheless, this study proposed the possibility of wild *P. yedoensis* from Jeju Island and cultivated *P.* × *yedoensis* being different taxonomic entities with independent origin.

In our study, all the accessions of *P. spachiana* f. *ascendens* (= *P. subhirtella* f. *ascendens*) except for four unusual accessions nested within the clade of *P. serrulata*/*P. sargentii*/*P*. *speciosa* complex were closely related to both wild *P. yedoensis* and cultivated *P.* × *yedoensis* based on cpDNA phylogeny of seven noncoding regions (**Figure 2**). Most chlorotypes of *P. spachiana* f. *ascendens* were shared between the plants of two regions (Japan and Jeju Island), while some occurred exclusively within one region (**Figure 5**). These results suggest that the individuals from Japan and Jeju Island sharing the same chlorotypes most likely contributed as maternal donors to the origin of both wild *P. yedoensis* on Jeju Island and cultivated *P.* × *yedoensis* in Japan. In the cpDNA haplotype network, all accessions of cultivated *P.* × *yedoensis* had the H10 haplotype, which they shared with only two out of 75 accessions of *P. spachiana* f. *ascendens* (one accession from Japan and one from the Korean peninsula). H10 haplotype, which corresponded to the “AA” haplotype from the study of Roh et al., did not include any accessions of *P. spachiana* f. *ascendens* collected from Jeju Island. The remaining *P. spachiana* f. *ascendens* accessions shared the “TA” haplotype with all except two accessions (YE833-1 and 833-2) of wild *P. yedoensis* that unexpectedly had the H10 haplotype. The observed haplotype patterns of *P. spachiana* f. *ascendens* suggest that the accessions having the “AA” haplotype of the *rpl*16 gene, which is relatively rare in natural populations, could be related to cultivated *P.* × *yedoensis* exclusively as a result of hybridization.

Nakamura et al. (2015) proposed that maternal species of Somei-yoshino is the cultivar “Komatsu-otome” or a related cultivar. This conclusion was based on the fact that the “Komatsu-otome” haplotype was identical to one of the Somei-yoshino haplotypes based on the *PolA1* 19 intron region, and this haplotype was different from *P. pendula* (= *P. spachiana* f. *ascendens*). In this research, we confirmed the presence of the same haplotype in the cultivated *P.* × *yedoensis* based on the same gene region at the same position as described in Nakamura et al. (2015) (position 430, **Table S6**). However, we found that *P. spachiana* f. *ascendens* accessions collected from natural population in Sendai region, Japan, had the same polymorphism as “Komatsu-otome.” Therefore, we suggest that the maternal parent of cultivated *P.* × *yedoensis* is most likely an individual or individuals of *P. spachiana* f. *ascendens* having both the same polymorphism of the *PolA1* 19 intron region and the “AA” haplotype of the *rpl*16 gene as “Komatsu-otome,” which is a combination rarely found in Japan. This maternal parent can be distinguished from *P. spachiana* f. *ascendens* by sharing variable haplotypes with wild *P. yedoensis* from Jeju Island. The selection of the specific and rare haplotype of *P. spachiana* f. *ascendens* in the hybrid origin of cultivated *P.* × *yedoensis* supports the hypothesis of artificial hybridization given by Iwasaki Fumio (1991). Two unusual accessions of wild *P. yedoensis*, 833-1 and 833-2, were nested within the H10 haplotype shared by cultivated *P.* × *yedoensis*. These two accessions were collected from the natural forest around Kwaneum Temple on Mt. Halla. This is the same location where three accessions of wild *P*. *yedoensis* (2, 3, and 5) with the “AA” haplotype (exclusive to cultivated *P.* × *yedoensis*) were collected by Roh et al. (2007). Another accession of wild *P. yedoensis* (408003) that we collected from the same population displayed the same type of SNPs as cultivated *P.* × *yedoensis* in the sequence analyses of RosCOS and *PolA1* data set (**Tables S5**, **S6**, and **S7**). Given this unexpected placement of wild accessions collected from the same population around Kwaneum Temple (reported independently in two separate studies), we suggest the possibility of cultivated flowering cherries (*P.* × *yedoensis*) having escaped from cultivation and spread into natural habitats including the forest surrounding Kwaneum Temple and adjacent areas, creating mixed stands of wild *P. yedoensis* and cultivated *P.* × *yedoensis*. As an alternative theory, cultivated *P.* × *yedoensis* might have been intentionally planted around the Temple or in the Hallasan National Park. Therefore, it is possible that the samples from Kwaneum Temple were collected and misidentified as wild *P*. *yedoensis* because of high morphological similarities between wild and cultivated flowering cherries and an unexpected locality for wild *P. yedoensis*. Wild *P. yedoensis* individuals are rarely found in undisturbed natural habitats surrounding Mt. Halla, while numerous cultivated *P.* × *yedoensis* plants are usually planted along streets and roads. Excluding the two unusual constituents of wild *P*. *yedoensis* accessions (833-1 and 833-2), the cpDNA haplotype network clearly separated the haplotypes among accessions of wild *P. yedoensis* and cultivated *P.* × *yedoensis*. Accessions of cultivated *P.* × *yedoensis* had only one haplotype (H10), while the accessions of wild *P. yedoensis* contained diverse yet closely related haplotypes (H1, H2, H5, H6, H7, and H8), except for the H3 haplotype, which was a result of a reverse direction of hybridization event with *P. spachiana* f. *ascendens* as the paternal and *P. sargentii*/*P. serrulata* complex as the maternal parent. In the haplotype network, the H9 haplotype comprising mostly *P. spachiana* f. *ascendens* accessions from Japan was nested between wild *P. yedoensis* and cultivated *P.* × *yedoensis*, making a genetic distinction between them.

Different cpDNA genetic materials observed between wild *P. yedoensis* and cultivated *P.* × *yedoensis* were corroborated by biparentally inherited nrDNA. The ITS phylogeny (**Figures 3A** and **3B**) included 82 cloned accessions of wild *P. yedoensis* and cultivated *P.* × *yedoensis*, which had two kinds of ITS ribotypes originating from two parental lineages shown in the APS: 45 cloned accessions were nested within the maternal lineage of *P. spachiana* f. *ascendens* and *P. yedoensis*, while the remaining 37 cloned accessions were nested within the paternal lineage of the *P. serrulata*/*P. sargentii*/*P*. *speciosa* complex. We wanted to further investigate how the 37 cloned accessions of wild *P. yedoensis* and cultivated *P.* × *yedoensis* nested within the paternal lineage were related to putative paternal species. Due to lack of good resolutions and strong support, no clear distinction could be made between wild *P. yedoensis* and cultivated *P.* × *yedoensis*. However, two subclades provided some insights into the paternal contribution from related congeneric species (**Figure 3B**). The subclade of cultivated *P.* × *yedoensis* included the cloned accessions of cultivated *P.* × *yedoensis*, *P. speciosa*, and presumably misidentified wild *P. yedoensis* (833-2), which suggested that the paternal parent of cultivated *P.* × *yedoensis* was *P. speciosa* endemic to the Izu Islands and Izu Peninsula, Japan. On the contrary, all but a few exceptional accessions of wild *P. yedoensis* ribotypes on Jeju Island shared their most recent common ancestor with other congeneric species, including *P. serrulata* var. *spontanea* and *P. serrulata* var. *quelpaertensis* which occur rarely and are sympatric with wild *P. yedoensis* from Jeju Island. Therefore, nrDNA ITS sequences provided an additional line of evidence that different paternal species contributed to the origin of wild *P. yedoensis* and cultivated *P.* × *yedoensis* (*P. serrulata* var. *spontanea*/*P. serrulata* var. *quelpaertensis* and *P. speciosa*, respectively). With regard to the paternal contributor of wild *P. yedoensis*, our earlier study (Cho et al., 2014) suggested that members of an unresolved sympatric species complex *P. serrulata*/*P. sargentii* complex were most likely the paternal parent. In this study, however, we provided evidence of the involvement of *P. sargentii*, a commonly occurring species, in the origin of wild *P. yedoensis* being less probable. Considering certain morphological characteristics of somewhat variable wild *P. yedoensis*, a greater involvement of *P. serrulata* var. *spontanea* and *P. serrulata* var. *quelpaertensis* seems apparent. For example, a typical corymb inflorescence type in wild *P. yedoensis* is most likely a trait inherited from *P. serrulata*, which has elongated corymb inflorescence, rather than *P. sargentii*, which has a very short peduncle bearing an umbel-like inflorescence.

The theory of independent origin of wild *P. yedoensis* and cultivated *P.* × *yedoensis* was further corroborated by the results of the analyses of nuclear DNA SNPs of RosCOS loci and single copy *PolA1* data sets. Four RosCOS amplified data sets as well as 26 RosCOS data sets revealed the hybridization pattern of APS in the sequences of wild *P. yedoensis* and cultivated *P.* × *yedoensis* (**Table S5**). Neighbor-net split graph based on four RosCOS loci also indicated that the investigated species are of independent hybrid origin and that they originated from three major groups: *P. spachiana* f. *ascendens*, *P. speciosa* from Japan, and *P. serrulata*/*P. sargentii* complex on Jeju Island (**Figure 4**). Hybrid accessions of wild *P. yedoensis* and cultivated *P.* × *yedoensis* were positioned in the middle of the graph linking three parental groups together without their own edges, which is the expected pattern in a recent or F_1_ hybrid. Furthermore, four RosCOS data sets showed that the presence of different paternal contributions was reflected in the abundance of species-specific sites shared by wild *P. yedoensis* and cultivated *P.* × *yedoensis* (sites 80, 133, 144, 229, 244, 295, 308, 449, 467, 793, 853, 1090, 1148, 1172, and 1215; **Table S5**).

Likewise, the *PolA1* data set indicated a hybridization pattern of APS in the sequences of wild *P. yedoensis* and cultivated *P.* × *yedoensis* as well as differentiated paternal parents between these species on intron 19 (sites 1, 61, 139, 209, 430, 492, and 495; **Table S6**) and PE20 exon region (sites 121, 463, 471, 534-557, 676, 705, and 712; **Table S7**). Based on SNPs of RosCOS and *PolA1* data sets, we observed that the accessions of wild *P. yedoensis* tended to show the admixture pattern of variable sequences, while the accessions of cultivated *P.* × *yedoensis* showed monomorphic sequences. The admixture sequence patterns in wild *P. yedoensis* are indicative of random and recurrent spontaneous hybridization events. Our current results have been independently confirmed based on different sets of 38 *Prunus*-specific COS markers (Cho et al., 2017). The results of those analyses supported our current findings on the hybrid origin of wild *P. yedoensis* and suggested that ca. 80% of the sampled accessions were most likely F_1_, while the remaining 20% represented additional generations of asymmetric introgression of the parental genotypes. Therefore, we provided convincing evidence for the independent origin of wild *P. yedoensis* and cultivated *P.* × *yedoensis*, cultivated *P.* × *yedoensis* being the artificial hybrid resulting from a cross between *P. spachiana* f. *ascendens* as the maternal parent and *P. speciosa* as the paternal parent, and wild *P. yedoensis* being a spontaneous hybrid between *P. spachiana* f. *ascendens* as the maternal parent and *P. serrulata* var. *spontanea* and *P. serrulata* var. *quelpaertensis* as the most likely paternal parent. Our current findings based on the phylogenetic and phylogeographic approach have been independently supported by the results of the whole genome sequencing of wild *P. yedoensis* (Baek et al., 2018) and cultivated *P.* × *yedoensis* “Somei-yoshino” (Shirasawa et al., 2019).

### Development of New Cultivars and Germplasm Conservation of Wild *P. yedoensis*

Wild populations of cultivated ornamental trees and shrubs are excellent sources of genetic material for the development of new cultivars as well as for special breeding programs for desirable traits and disease resistance. Out of approximately 250 species of the genus *Prunus*, the most important and traditional group used as ornamentals are the flowering cherries of subg. *Cerasus* sect. *Pseudocerasus*, which include a small group trees with spectacular flowers native to eastern Asia (Rehder, 1940). Although these ornamental flowering cherry trees are important ornamental plants throughout the world, the most widely cultivated flowering cherry trees are descendants of only a few species, and given their clonal propagation, they are highly susceptible to several serious pest problems and diseases (Pscheidt and Byther, 2001). Various breeding programs of major institutes, such as the United States National Arboretum, are aiming at developing new cultivars with disease and pest resistance, tolerance to environmental stresses, and superior ornamental characteristics (Pooler et al., 2012). However, such breeding practices in the genus *Prunus* face unique challenges because of the narrow genetic background of commercial cultivars, a long juvenile period, large plant size, and differences in trait expression between juvenile and mature trees (Scorza et al., 1985; Baird et al., 1996; van Nocker and Gardiner, 2014). Recently, traditional breeding practices have been advanced by the application of new biological tools and biotechnologies, including *in vitro* tissue culture, genetic transformation, molecular maker development, and cryopreservation (Cheong, 2012).

**Figure 6:**
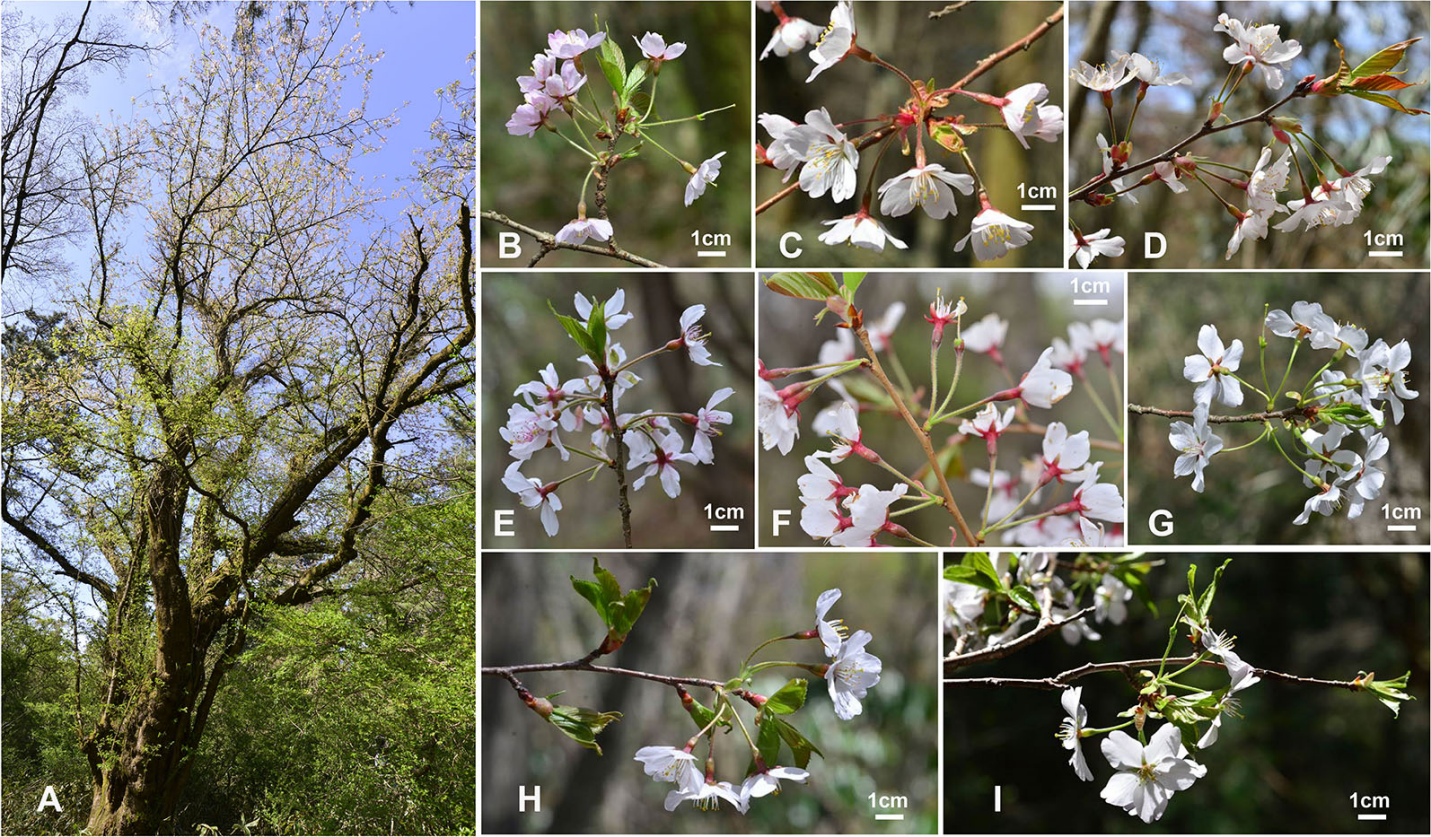
Variation in plant morphology between natural populations of *P. yedoensis* on Jeju Island, Korea. **(A)** The oldest known wild *P. yedoensis* individual with the estimated age of 265 years and the height and girth of 15.5 m and 4.5 m, respectively. **(B**–**I)** Morphological variation observed in wild *P. yedoensis* driven by multiple bidirectional hybridization and introgression events. (Photo credit: Gwan Pil Song, Jeju Biological Resource).

We believe that wild populations of flowering cherries, especially wild *P. yedoensis* naturally occurring on Jeju Island, can be important sources of raw genetic material in future breeding programs. This study as well as our previous study (Cho et al., 2014) and some independent studies (Baek et al., 2018; Cho et al., 2018) demonstrated that wild *P. yedoensis* is of hybrid origin resulting from multiple bidirectional hybridization events between *P. spachiana* f. *ascendens* (as the maternal parent) and *P. serrulata*/*P. sargentii* complex (as the paternal parent). Although these two genetically divergent parental lineages were determined to be the ones most likely involved in the origin of wild *P. yedoensis*, the potential involvement of other sympatric congeneric species from Jeju Island was not completely ruled out because of insufficient distinction in their phylogenetic relationships. Nevertheless, the combination of F_1_ hybrids (ca. 80%) and backcrossing introgressants (ca. 20%) resulted in diverse morphological variations in plants from Jeju Island (Cho et al., 2017) (**Figure 6B-1**). Furthermore, reverse direction of hybridization (*P. spachiana* f. *ascendens* as the paternal parent and *P. serrulata*/*P. sargentii* as the maternal parent) and subsequent backcrossing introgression events also contributed to the origin of *P. yedoensis* on Jeju Island, increasing the level of morphological and genetic variations (Cho et al., 2014). Unlike in genetically uniform cultivated *P.* × *yedoensis* “Somei-yoshino,” wild populations on Jeju Island have diverse morphological and genetic variations and they have managed to bypass the long juvenile stage and increase their genetic variability (that is usually low in cultivated *Prunus* species). Since 2016, a total of 194 individuals of wild *P. yedoensis* have been reported primarily in northern and eastern parts of Jeju Island (Jeju Biological Resources, 2016). These wild accessions can be subjected to selection and breeding programs for the development of new cultivars and improvement of existing commercial cultivars for superior horticultural traits. For example, unlike the typical cultivated *P.* × *yedoensis* “Somei-yoshino” plant that has a short lifespan of 25–50 years (although there are many trees well over 100 years old), a wild approximately 265 years old *P. yedoensis* plant was discovered in the northeastern part of Hallasan Mountain National Park (Bonggae-dong, Gaeoleum cinder cone)^2^ (**Figure 6A**). Along with another individual designated as Natural Monument #158 in Bonggae-dong (estimated age of 200 years), these wild individuals can be used for selecting for longevity and any other desirable traits through breeding programs. To achieve this goal, it is critical to carefully characterize and document morphological and genetic variations of wild populations found throughout Jeju Island and to screen them for potential disease and pest resistance traits as well as various other horticultural traits.

More importantly, wild *P. yedoensis* on Jeju Island is currently listed as an endangered species (ER) according to the IUCN categories (i.e., very narrow geographical distribution and small number of individuals) (NIBR, 2014). Most wild *P. yedoensis* individuals are typically found in undisturbed natural habitats, e.g., ravines surrounding Mt. Halla (Cho et al., 2014). However, some individuals occur along the streets and roads or on forest edges and are exposed to potential anthropogenic disturbance. In addition, because of the lack of strong isolation mechanisms between congeneric species of subg. *Cerasus* and post-hybridization gene flow between wild *P. yedoensis* and its parental species, the fate of wild *P. yedoensis* remains uncertain in terms of conservation. The two parental lineages of wild *P. yedoensis* appear to be genetically divergent, although without strong reproductive isolation mechanisms. Based on their overlapping phenology, we concluded that a hybridization event that occurred between them resulted in the formation of sterile wild *P. yedoensis*. Although many natural hybrids are sterile, some hybrid individuals can be fertile and involved in later hybridization events. These complex evolutionary processes may result in generations abundant with admixture individuals sympatrically occurring with congeneric parental species, and this could further facilitate the ongoing gene flow process. For conservation purposes, it is critical to carefully document and characterize these complex ongoing evolutionary processes and genetic reservoirs, to identify any natural and anthropogenic threatening factors, and to establish and implement conservation strategies and management plans.

## Data Availability Statement

All datasets generated for this study are available at Dryad under doi: 10.5061/dryad.cz8w9gj02.

## Author Contributions

M-SC and S-CK designed the experiment and collected the samples. M-SC performed the experiments and analyzed the data. M-SC drafted the manuscript and S-CK revised it. M-SC and S-CK both approved the final manuscript.

## Funding

This project was supported by the Basic Science Research Program through the National Research Foundation of Korea (NRF) (grant numbers 2017R1A6A3A01075954 and 2019R1A6A1A10073079).

## Conflict of Interest

The authors declare that the research was conducted without any commercial or financial support that could be construed as a potential conflict of interest.

## Footnotes

1
http://www.rosaceae.org/species/rosaceae_family_genera/RosCOS

2
https://www.yna.co.kr/view/AKR20160503055400056?input=1195m
