# Supplementary material for: Multiple Lines of Evidence for Independent Origin of Wild and Cultivated Flowering Cherry (Prunus yedoensis)
Source: Front Plant Sci. 2019 Dec 19;10:1555. doi: 10.3389/fpls.2019.01555 (PMC6930925; doi:10.3389/fpls.2019.01555)

Supplementary Material

Multiple lines of evidence for independent origin of wild and cultivated flowering cherry (*Prunus yedoensis*)

Myong-Suk Cho and Seung-Chul Kim^*^

*** Correspondence**: Seung-Chul Kim: sonchus96@skku.edu

# Supplementary Figure and Tables

## 1.2 Supplementary Tables

**Supplementary Table S1**. The list of plant materials used for multiple phylogenetic analyses in this study.

| Species/  Accession No. | Collection Site | | Collection  Date | Specimen No. | Analyses used for this study | | | |
| --- | --- | --- | --- | --- | --- | --- | --- | --- |
|  |  |  |  |  | ITS/ETS/  7 cpDNA regions  Phylogeny | cpDNA Network | ROSCOS  SNP | POLA1  SNP |
| **Outgroup, Subgenus *Padus,* Genus *Prunus*** | | | | | | | | |
| ***Prunus padus* L.** | | | | | | | | |
| 907 | Mooju, Korea | | 13-May-2011 | *SKK Cho et al. 11513169* | О |  |  |  |
| ***Prunus grayana* Maxim.** | | | | | | | | |
| 13054 | Aomori, Japan | | 10-May-13 | *SKK Cho et al.13054* | О |  |  |  |
| ***Prunus buergeriana* Miq.** | | | | | | | | |
| 13045 | Fukushima, Japan | | 2-May-13 | *SKK Cho et al. 13045* | О |  |  |  |
| 93 | Seongpanak Rest Area, Jeju | | 11-May-12 | *SKK Cho et al. 120511170* | О |  |  |  |
| **Ingroup, Subgenus *Cerasus,* Genus *Prunus*** | | | | | | | | |
| ***Prunus maximowiczii* Ruprecht collected from Jeju Island, Korea, section *Phyllomahaleb* Koehne** | | | | | | | | |
| 82_1 | Witseoreum | | 09-May-12 | *SKK Cho et al. 120509164* | О |  |  |  |
| 82_5 | Witseoreum | | 09-May-12 | *SKK Cho et al.120509165* | О |  |  |  |
| 876 | Bangseongyo | | 03-May-11 | *SKK Cho et al. 110503163* | О |  |  |  |
| ***Prunus spachiana*f.*ascendens*(Makino) Kitam. collected from Jeju Island, Korea, section *Pseudocerasus*** Koehne | | | | | | | | |
| 10 | Dooryunsan Wangbeot Natural Stand | | 17-Apr-12 | *SKK Cho et al. 120417124* | О | О |  |  |
| 11_1 | Dooryunsan Wang Beot Natural Stand | | 17-Apr-12 | *SKK Cho et al. 120417125* | О | О |  |  |
| 422004 | Bonggae Wangbeot Natural Stand | | 22-Apr-12 | *SKK Cho et al. 120422004* | О | О | О | О |
| 511004 | Youngpyung-dong, Jeju | | 11-May-12 | *SKK Cho et al. 120511004* | О | О |  |  |
| 511005 | Youngpyung-dong, Jeju | | 11-May-12 | *SKK Cho et al. 120511005* | О | О |  |  |
| 511006 | Youngpyung-dong, Jeju | | 11-May-12 | *SKK Cho et al. 120511006* | О | О |  |  |
| 1 | Sinrye-ri, Wangbeot Natural Stand | | 13-Apr-11 | *SKK Cho et al. 110413123* | О | О |  |  |
| 809 | Mysterious Road | | 12-Apr-11 | *SKK Cho et al. 110412126* | О | О | О | О |
| 815 | Hannam-ri | | 13-Apr-11 | *SKK Cho et al. 110413127* | О | О |  |  |
| 817 | Pyoseon-ri | | 14-Apr-11 | *SKK Cho et al. 110414128* | О | О | О | О |
| 820 | Jedong Ranch | | 14-Apr-11 | *SKK Cho et al. 110414129* | О | О |  |  |
| 821 | Bonggae-dong | | 14-Apr-11 | *SKK Cho et al. 110414130* | О | О |  |  |
| 827 | Seongpangyo | | 18-Apr-11 | *SKK Cho et al. 110418131* | О | О |  |  |
| 830_1 | Beopjung-dong | | 18-Apr-11 | *SKK Cho et al. 130418200* | О | О |  |  |
| 830_K13 | Beopjung-dong | | 18-Apr-11 | *SKK Cho et al. 130418201* | О | О |  |  |
| 830_K6 | Beopjung-dong | | 18-Apr-11 | *SKK Cho et al. 130428202* | О | О |  |  |
| 831 | Beopjung-sa | | 18-Apr-11 | *SKK Cho et al. 110418232* | О | О |  |  |
| 832 | Kwaneumsa | | 19-Apr-11 | *SKK Cho et al. 110419133* | О | О |  |  |
| 833_10 | Kwaneumsa | | 19-Apr-11 | *SKK Cho et al. 130419200* | О | О |  |  |
| 833_14 | Kwaneumsa | | 19-Apr-11 | *SKK Cho et al. 130419201* | О | О |  |  |
| 833_22 | Kwaneumsa | | 19-Apr-11 | *SKK Cho et al. 130419202* | О | О | О | О |
| 833_8 | Kwaneumsa | | 19-Apr-11 | *SKK Cho et al. 130419203* |  | О | О | О |
| 857 | Youngsil | | 02-May-11 | *SKK Cho et al. 110502134* | О | О |  |  |
| 859_038 | 1100 Rest area | | 02-May-11 | *SKK Cho et al. 130502200* | О | О |  |  |
| 866_003 | Seoguipo Natural Recreation Forest | | 02-May-11 | *SKK Cho et al. 130502201* | О | О |  |  |
| 877 | Eorimok | | 03-May-11 | *SKK Cho et al. 110503135* | О | О |  |  |
| ***Prunus spachiana*f.*ascendens*(Makino) Kitam. collected from Japan, section *Pseudocerasus* Koehne** | | | | | | | | |
| 377_006 | Sendai | | 22-Apr-13 | *SKK Cho et al. 130422200* | О | О |  |  |
| 378_012 | Sendai | | 22-Apr-13 | *SKK Cho et al. 130422201* | О | О | О | О |
| 378_016 | Sendai | | 22-Apr-13 | *SKK Cho et al. 130422202* | О | О |  |  |
| 378_008 | Sendai | | 22-Apr-13 | *SKK Cho et al. 130422203* |  | О |  |  |
| 378_013 | Sendai | | 22-Apr-13 | *SKK Cho et al. 130422204* |  | О |  |  |
| 378_018 | Sendai | | 22-Apr-13 | *SKK Cho et al. 130422205* |  | О | О | О |
| 382_034 | Sendai | | 22-Apr-13 | *SKK Cho et al. 130422206* | О | О |  |  |
| 382_039 | Sendai | | 22-Apr-13 | *SKK Cho et al. 130422207* | О | О | О | О |
| 390_025 | Miyagi | | 24-Apr-13 | *SKK Cho et al. 130424200* | О | О | О | О |
| 395_048 | Miyagi | | 24-Apr-13 | *SKK Cho et al. 130424201* | О | О |  |  |
| 395_049 | Miyagi | | 24-Apr-13 | *SKK Cho et al. 130424202* | О | О | О | О |
| 927_102 | Hiroshima | | 03-Apr-14 | *SKK Cho et al. 140403200* |  | О |  |  |
| 1TOHO | Tohoku Univ. Botanical Garden, Sendai | | 20-Apr-12 | None | О | О |  |  |
| 2_1MARU | Miyagi | | 29-Apr-12 | None | О | О |  |  |
| 2_2MARU | Miyagi | | 29-Apr-12 | None | О | О |  |  |
| 3TOHO | Tohoku Univ. Botanical Garden, Sendai | | 08-May-12 | None | О | О |  |  |
| 517_1 | Yamagata | | 17-May-15 | None |  | О |  |  |
| 706_2 | Inagawa | | 06-Jul-15 | None |  | О |  |  |
| 706_7 | Inagawa | | 06-Jul-15 | None |  | О |  |  |
| 706_10 | Inagawa | | 06-Jul-15 | None |  | О |  |  |
| 706_16 | Inagawa | | 06-Jul-15 | None |  | О |  |  |
| 706_20 | Inagawa | | 06-Jul-15 | None |  | О |  |  |
| 706_22 | Inagawa | | 06-Jul-15 | None |  | О |  |  |
| 706_1 | Inagawa | | 06-Jul-15 | None |  | О |  |  |
| 706_11 | Inagawa | | 06-Jul-15 | None |  | О |  |  |
| 706_12 | Inagawa | | 06-Jul-15 | None |  | О |  |  |
| 706_13 | Inagawa | | 06-Jul-15 | None |  | О |  |  |
| 706_14 | Inagawa | | 06-Jul-15 | None |  | О |  |  |
| 706_15 | Inagawa | | 06-Jul-15 | None |  | О |  |  |
| 706_17 | Inagawa | | 06-Jul-15 | None |  | О |  |  |
| 706_18 | Inagawa | | 06-Jul-15 | None |  | О |  |  |
| 706_19 | Inagawa | | 06-Jul-15 | None |  | О |  |  |
| 706_21 | Inagawa | | 06-Jul-15 | None |  | О |  |  |
| 706_3 | Inagawa | | 06-Jul-15 | None |  | О | О | О |
| 706_4 | Inagawa | | 06-Jul-15 | None |  | О |  |  |
| 706_5 | Inagawa | | 06-Jul-15 | None |  | О |  |  |
| 706_6 | Inagawa | | 06-Jul-15 | None |  | О |  |  |
| 706_8 | Inagawa | | 06-Jul-15 | None |  | О |  |  |
| 706_9 | Inagawa | | 06-Jul-15 | None |  | О |  |  |
| ***Prunus yedoensis* M., wild and endemic to Jeju Island, Korea, section *Pseudocerasus* Koehne** | | | | | | | | |
| 422003 | Bonggae Wangbeot Natural Stand | | 22-Apr-12 | *SKK Cho et al. 120422200* | О | О | О | О |
| 5191000 | Bonggae-dong | | 19-May-15 | *SKK Cho et al. 150519200* |  | О | О | О |
| 408003 | Kwaneumsa | | 08-Apr-16 | *SKK Cho et al. 160408200* |  |  | О | О |
| 3_1 | Haryeri, Wangbeot Natural Stand | | 13-Apr-11 | *SKK Cho et al. 110413100* | О | О |  |  |
| 3_A | Haryeri, Wangbeot Natural Stand | | 13-Apr-11 | *SKK Cho et al. 110413101* | О | О | О | О |
| 3_B | Haryeri, Wangbeot Natural Stand | | 13-Apr-11 | *SKK Cho et al. 110413102* | О | О |  |  |
| 804 | Haean-dong | | 12-Apr-11 | *SKK Cho et al. 110411200* |  | О |  |  |
| 814 | Wimi-ri | | 13-Apr-11 | *SKK Cho et al. 110413105* | О | О |  |  |
| 816 | Hannam-ri | | 14-Apr-11 | *SKK Cho et al. 110414200* |  | О |  |  |
| 826_1 | Bonggae Wangbeot Natural Stand | | 13-Apr-11 | *SKK Cho et al. 110413106* | О | О | О | О |
| 826_2 | Bonggae Wangbeot Natural Stand | | 13-Apr-11 | *SKK Cho et al. 110413107* | О | О | О | О |
| 830_1 | Beopjung-dong | | 18-Apr-11 | *SKK Cho et al. 110418200* |  | О | О | О |
| 833_1 | Kwaneumsa | | 19-Apr-11 | *SKK Cho et al. 110419200* |  | О | О | О |
| 833_2 | Kwaneumsa | | 19-Apr-11 | *SKK Cho et al. 110419108* | О | О |  |  |
| 836_9 | 1^st^ Sallok Rd. | | 19-Apr-11 | *SKK Cho et al. 110419201* |  | О |  |  |
| 836_1 | 2^nd^ Sallok Rd. | | 19-Apr-11 | *SKK Cho et al. 110419202* |  | О |  |  |
| 857_34 | Youngsil | | 02-May-11 | *SKK Cho et al. 110502200* |  | О |  |  |
| 867_19 | Sinrye-ri, Wangbeot Natural Stand | | 03-May-11 | *SKK Cho et al. 110503200* |  | О |  |  |
| 868 | Sinrye-ri, Wangbeot Natural Stand | | 03-May-11 | *SKK Cho et al. 110503109* | О | О | О | О |
| 871 | Hannam-dong | | 03-May-11 | *SKK Cho et al. 110503201* |  | О |  |  |
| 872 | Seongpangyo | | 03-May-11 | *SKK Cho et al. 110503110* | О | О |  |  |
| 873 | Kwaneumsa | | 03-May-11 | *SKK Cho et al. 110503111* | О | О |  |  |
| 875 | Tamra Education Center | | 03-May-11 | *SKK Cho et al. 110503112* | О | О |  |  |
| 876 | Bangseongyo | | 03-May-11 | *SKK Cho et al. 110503113* | О | О | О | О |
| 879 | Eorimok | | 03-May-11 | *SKK Cho et al. 110503114* | О | О |  |  |
| 880 | Eorimok | | 03-May-11 | *SKK Cho et al. 110503115* | О | О | О | О |
| 92 | Daepo-dong | | 10-May-12 | *SKK Cho et al. 120510104* | О | О | О |  |
| 94_3 | Youngpyeong-dong, Jeju | | 11-May-12 | *SKK Cho et al. 120511200* |  | О |  |  |
| 9 | Dooryunsan, Wangbeot Natural Stand | | 17-Apr-12 | *SKK Cho et al. 120417103* | О | О | О | О |
| **Cultivated *Prunus* ×*yedoensis* M., collected from Japan and Korea, section *Pseudocerasus* Koehne** | | | | | | | | |
| 808_5 | Jeju University | | 12-Apr-11 | *SKK Cho et al. 110412116* | О | О |  |  |
| 808_8 | Jeju University | | 12-Apr-11 | *SKK Cho et al. 110412117* | О | О |  |  |
| JINHAE1 | Jinhae | | 29-Apr-12 | *SKK Cho et al. 120429118* | О | О |  |  |
| JINHAE3 | Jinhae | | 29-Apr-12 | *SKK Cho et al. 120429119* | О | О |  |  |
| JINHAE5 | Jinhae | | 29-Apr-12 | *SKK Cho et al. 120429120* | О | О |  |  |
| SNU_K | Seoul National University | | 26-Apr-12 | *SKK Cho et al. 120426121* | О | О |  |  |
| SNU_N | Seoul National University | | 26-Apr-12 | *SKK Cho et al. 20426122* | О | О |  |  |
| KO3492 | Koishikawa Botanical Garden, Tokyo | | 18-Apr-13 | None | О | О | О | О |
| KO4972 | Koishikawa Botanical Garden, Tokyo | | 18-Apr-13 | None | О | О | О | О |
| KO4981 | Koishikawa Botanical Garden, Tokyo | | 18-Apr-13 | None | О | О | О | О |
| KO5033 | Koishikawa Botanical Garden, Tokyo | | 18-Apr-13 | None | О | О | О | О |
| KO5041 | Koishikawa Botanical Garden, Tokyo | | 18-Apr-13 | None | О | О | О | О |
| ***Prunus sargentii* Rehder collected from Jeju Island, Korea, section *Pseudocerasus* Koehne** | | | | | | | | |
| 833_A29 | Kwaneumsa | | 19-Apr-11 | *SKK Cho et al. 110419138* | О |  | О |  |
| 834 | 1^st^ Sallok Rd. | | 19-Apr-11 | *SKK Cho et al. 110419139* | О |  | О |  |
| 836_4 | 1^st^ Sallok Rd. | | 19-Apr-11 | *SKK Cho et al. 110419140* | О |  | О |  |
| 837 | Sancheondan | | 19-Apr-11 | *SKK Cho et al. 110419141* | О |  | О |  |
| 838_4 | Mysterious Road | | 19_Apr-11 | *SKK Cho et al. 110419203* |  |  | О |  |
| 857_30 | Youngsil | | 02-May-11 | *SKK Cho et al. 110502142* |  |  | О |  |
| 861 | 1100 Rest area | | 02-May-11 | *SKK Cho et al. 110502201* | О |  | О | О |
| 862_10 | 1100 Rest area | | 02-May-11 | *SKK Cho et al. 110502202* |  |  | О |  |
| 863_48 | Eorimok | | 02-May-11 | *SKK Cho et al. 110502143* | О |  | О |  |
| 866_5 | Seogwipo Natural Recreation Forest | | 02-May-11 | *SKK Cho et al. 110502203* |  |  | О | О |
| 868_8 | Sinrye-ri, Wangbeot Natural Stand | | 03-May-11 | *SKK Cho et al. 110503144* | О |  |  |  |
| 868_9 | Sinrye-ri, Wangbeot Natural Stand | | 03-May-11 | *SKK Cho et al. 110503145* | О |  |  |  |
| 869 | Harye-ri, Wangbeot Natural stand | | 03-May-11 | *SKK Cho et al. 110503146* | О |  | О |  |
| ***Prunus sargentii* Rehder collected from Korean Peninsula, section *Pseudocerasus* Koehne** | | | | | | | | |
| 511020 | Seoraksan | | 11-May-12 | *SKK Cho et al. 120511020* | О |  |  |  |
| 517001 | Hambaiksan | | 17-May-13 | *SKK Cho et al. 130517001* | О |  |  |  |
| 517002 | Hambaiksan | | 17-May-13 | *SKK Cho et al. 130517002* | О |  |  |  |
| 430014 | Minjujisan | | 30-Apr-14 | *SKK Cho et al. 140430014* | О |  |  |  |
| 501006 | Deogyusan | | 01-May-14 | *SKK Cho et al. 140501006* | О |  |  |  |
| 501500 | Deogyusan | | 01-May-14 | *SKK Cho et al. 140501500* | О |  |  |  |
| 425100 | Jukryeong, Sobaeksan | | 25-Apr-15 | *SKK Cho et al. 150425100* | О |  |  |  |
| 425110 | Sobaeksan | | 25-Apr-15 | *SKK Cho et al. 150425110* | О |  |  |  |
| 425112 | Sobaeksan | | 25-Apr-15 | *SKK Cho et al. 150425112* | О |  |  |  |
| 355_52 | Jirisan | | 12-Apr-13 | *SKK Cho et al. 130412052* | О |  |  |  |
| 355_54 | Jirisan | | 12-Apr-13 | *SKK Cho et al. 130412054* | О |  |  |  |
| 42_112 | Odaesan | | 10-May-14 | *SKK Cho et al. 140510112* | О |  |  |  |
| 44_19 | Odaesan | | 10-May-14 | *SKK Cho et al. 140510019* | О |  |  |  |
| 44_21 | Odaesan | | 10-May-14 | *SKK Cho et al. 140510021* | О |  |  |  |
| ***Prunus sargentii* Rehder collected from Russia, section *Pseudocerasus* Koehne** | | | | | | | | |
| 712006RS | Botanical Garden Institute FEB RAS | | 12-Jul-14 | *SKK Cho et al. 140712006* | О |  |  |  |
| 0708RS | Khansanskii | | 08-Jul-15 | *SKK Cho et al. 150708* | О |  |  |  |
| 803001RS | Chekhovo | | 03-Aug-15 | *SKK Cho et al. 150803001* | О |  |  |  |
| 803002RS | Chekhovo | | 03-Aug-15 | *SKK Cho et al. 150803002* | О |  |  |  |
| 803003RS | Chekhovo | | 03-Aug-15 | *SKK Cho et al. 150803003* | О |  |  |  |
| ***Prunus sargentii* Rehder collected from Japan, section *Pseudocerasus*** **Koehne** | | | | | | | | |
| 1TOHO | Tohoku Univ. Botanical Garden, Sendai | | 20-Apr-12 | None | О |  |  |  |
| 2_1HIGA | Higashikatsuyama, Sendai | | 26-Apr-12 | None | О |  |  |  |
| 2_2HIGA | Higashikatsuyama, Sendai | | 27-Apr-12 | None | О |  |  |  |
| 3MARU | Miyagi | | 29-Apr-12 | None | О |  |  |  |
| 383_1 | Miyagi | | 23-Apr-13 | *SKK Cho et al. 130423001* | О |  |  |  |
| 383_8 | Miyagi | | 23-Apr-13 | *SKK Cho et al. 130423008* | О |  |  |  |
| 928_200 | Okayama | | 03-Apr-14 | *SKK Cho et al. 140403200* | О |  |  |  |
| 932_301 | Okayama | | 03-Apr-14 | *SKK Cho et al. 140403301* | О |  |  |  |
| ***Prunus serrulata*var.*spontanea*(Maxim.) E.H.Wilson collected from Jeju Island, Korea, section *Pseudocerasus* Koehne** | | | | | | | | |
| 802 | Dosun-dong | | 12-Apr-11 | *SKK Cho et al. 110412147* | О |  |  |  |
| 26_19 | Namwon-eup | | 23-Apr-12 | *SKK Cho et al. 120423019* |  |  | О |  |
| 816_1_D7 | Hannam-ri | | 13-Apr-11 | *SKK Cho et al. 110413148* | О |  | О | О |
| 837 | Sancheondan | | 19-Apr-11 | *SKK Cho et al. 110419149* | О |  | О |  |
| 857_41 | Youngsil | | 02-May-11 | *SKK Cho et al. 110502041* |  |  | О |  |
| 866_12 | Seogwipo Natural Recreation Forest | | 02-May-11 | *SKK Cho et al. 110502150* | О |  | О | О |
| 878 | Young-dong, Jeju | | 03-May-11 | *SKK Cho et al. 110503151* | О |  | О | О |
| ***Prunus serrulata*var.*spontanea*(Maxim.) E.H.Wilson collected from Japan, section *Pseudocerasus* Koehne** | | | | | | | | |
| 1_1MARU | Miyagi | | 29-Apr-12 | None | О |  |  |  |
| 1_2MARU | Miyagi | | 29-Apr-12 | None | О |  |  |  |
| 1_3MARU | Miyagi | | 29-Apr-12 | None | О |  |  |  |
| 1_4MARU | Miyagi | | 29-Apr-12 | None | О |  |  |  |
| 2TOHO | Tohoku Univ. Botanical Garden, Sendai | | 08-May-12 | None | О |  |  |  |
| ***Prunus serrulata var. quelpaertensis* (Nakai) Uyeki collected from Jeju Island, Korea, section *Pseudocerasus* Koehne** | | | | | | | | |
| 3 | Haryeri Wangbeot Natural Stand | | 13-Apr-11 | *SKK Cho et al. 110413152* | О |  | О |  |
| 19_003 | Hannam-ri | | 23-Apr-12 | *SKK Cho et al. 120423003* |  |  | О |  |
| 23_012 | Seongpangyo | | 23-Apr-12 | *SKK Cho et al. 120423012* |  |  | О |  |
| 32_011 | Hanbalgyo | | 24-Apr-12 | *SKK Cho et al. 120424011* |  |  | О |  |
| 829 | Hannam Experimental Forest | | 18-Apr-11 | *SKK Cho et al. 110418154* | О |  |  |  |
| 834 | 1^st^ Sallok Rd. | | 19-Apr-11 | *SKK Cho et al. 110419155* | О |  |  |  |
| 838 | Sancheondan | | 19-Apr-11 | *SKK Cho et al. 110419156* | О |  | О | О |
| 860_25 | 1100 Resting Area | | 02-May-11 | *SKK Cho et al. 110502157* | О |  | О |  |
| 866_45 | Seogwipo Natural Recreational Forest | | 02-May-11 | *SKK Cho et al. 110502158* | О |  | О |  |
| 96_009 | Youngyyeong-dong | | 11-May-12 | *SKK Cho et al. 120511009* |  |  | О |  |
| ***Prunus serrulata* var. *pubescens* (Makino) collected from Korean Peninsula, section *Pseudocerasus* Koehne** | | | | | | | | |
| 9_0 | Dooryunsan Wang Beot Natural Stand | | 17-Apr-12 | *SKK Cho et al. 120417136* | О |  |  |  |
| 9_6 | Dooryunsan Wang Beot Natural Stand | | 17-Apr-12 | *SKK Cho et al. 120417153* | О |  |  |  |
| 10_2 | Dooryunsan Wang Beot Natural Stand | | 17-Apr-12 | *SKK Cho et al. 120417137* | О |  | О | О |
| ***Prunus serrulata* var. *pubescens* (Makino) collected from Japan, section *Pseudocerasus* Koehne** | | | | | | | | |
| 1TOHO | Tohoku Univ. Botanical Garden, Sendai | | 08-May-12 | None | О |  |  |  |
| ***Prunus yedoensis* M. *var. angustipetala* Kim *et* Kim collected from Jeju Island, Korea, section *Pseudocerasus*** **Koehne** | | | | | | | | |
| 832 | Kwaneumsa | | 19-Apr-11 | *SKK Cho et al.110419159* | О |  |  |  |
| ***Prunus longistylus* Kim *et* Kim collected from Jeju Island, Korea, section *Pseudocerasus* Koehne** | | | | | | | | |
| 874 | Tamra Valley | | 03-May-11 | *SKK Cho et al.110503162* | О |  |  |  |
| ***Prunus hallasanensis* Kim *et* Kim collected from Jeju Island, Korea, section *Pseudocerasu* Koehne** | | | | | | | | |
| 91 | Ara Sameuiak Trekking course | | 10-May-12 | *SKK Cho et al. 120510161* | О |  |  |  |
| 829 | Hannam Experimental Forest | | 18-Apr-11 | *SKK Cho et al. 110418160* | О |  |  |  |
| ***Prunus speciosa* (Koidz.) Ingram collected from Korea, cultivated, section *Pseudocerasus* Koehne** | | | | | | | | |
| 88 | Gimnyeong-ri, Jeju Island | | 10-May-12 | *SKK Cho et al. 120510167* | О |  |  |  |
| 89 | Gimnyeong-ri, Jeju Island | | 10-May-12 | *SKK Cho et al. 120510168* | О |  |  |  |
| KNR | Gimnyeong-ri, Jeju Island | | 25-Oct-11 | *SKK Cho et al. 111025166* | О |  |  |  |
| ***Prunus speciosa* (Koidz.) Ingram collected from Japan, section *Pseudocerasus* Koehne** | | | | | | | | |
| KO89_00277 | Koishikawa Botanical Garden, Tokyo | | 18-Apr-13 | None | О |  | О |  |
| KO89_251 | Koishikawa Botanical Garden, Tokyo | | 18-Apr-13 | None | О |  | О |  |
| 1TOHO | Tohoku Univ. Botanical Garden, Sendai | | 20-Apr-12 | None | О |  |  |  |
| 0424_065 | Tohoku Univ. Botanical Garden, Sendai | | 24-Apr-13 | *SKK Cho et al. 130424065* | О |  |  |  |
| 0424_066 | Tohoku Univ. Botanical Garden, Sendai | | 24-Apr-13 | *SKK Cho et al. 130424066* | О |  |  |  |
| 0425_014 | Tohoku Univ. Botanical Garden, Sendai | | 25-Apr-13 | *SKK Cho et al. 130425014* | О |  |  |  |
| 363_007 | Hakone | | 20-Apr-13 | *SKK Cho et al. 130420007* | О |  |  |  |
| 363_013 | Hakone | | 20-Apr-13 | *SKK Cho et al. 130420013* | О |  |  |  |
| 320006 | Oshima Island | | 20-Mar-15 | *SKK Cho et al. 150320006* |  |  | О |  |
| 320019 | Oshima Island | | 20-Mar-15 | *SKK Cho et al. 150320019* |  |  | О |  |
| 320036 | Oshima Island | | 20-Mar-15 | *SKK Cho et al. 150320036* |  |  | О |  |
| 320043 | Oshima Island | | 20-Mar-15 | *SKK Cho et al. 150320043* |  |  | О |  |
| 320047 | Oshima Island | | 20-Mar-15 | *SKK Cho et al. 150320047* |  |  | О |  |
| 320053 | Oshima Island | | 20-Mar-15 | *SKK Cho et al. 150320053* |  |  | О |  |
| 320067 | Oshima Island | | 20-Mar-15 | *SKK Cho et al. 150320067* |  |  | О |  |
| 320079 | Oshima Island | | 20-Mar-15 | *SKK Cho et al. 150320079* |  |  | О |  |
| 320085 | Oshima Island | | 20-Mar-15 | *SKK Cho et al. 150320085* |  |  | О |  |
| 320087 | Oshima Island | | 20-Mar-15 | *SKK Cho et al. 150320087* |  |  | О |  |
| 320012 | Oshima Island | | 20-Mar-15 | *SKK Cho et al. 150320012* | О |  | О | О |
| 320028 | Oshima Island | | 20-Mar-15 | *SKK Cho et al. 150320028* | О |  | О |  |
| 320031 | Oshima Island | | 20-Mar-15 | *SKK Cho et al. 150320031* | О |  | О |  |
| 320060 | Oshima Island | | 20-Mar-15 | *SKK Cho et al. 150320060* | О |  | О | О |
| 321002 | Kozushima Island | | 21-Mar-15 | *SKK Cho et al. 150321002* |  |  | О |  |
| 321023 | Kozushima Island | | 21-Mar-15 | *SKK Cho et al. 150321023* |  |  | О |  |
| 321026 | Kozushima Island | | 21-Mar-15 | *SKK Cho et al. 150321026* |  |  | О |  |
| 321038 | Kozushima Island | | 21-Mar-15 | *SKK Cho et al. 150321038* |  |  | О |  |
| 321062 | Kozushima Island | | 21-Mar-15 | *SKK Cho et al. 150321062* |  |  | О |  |
| 321028 | Kozushima Island | | 21-Mar-15 | *SKK Cho et al. 150321028* | О |  | О | О |
| 321042 | Kozushima Island | | 21-Mar-15 | *SKK Cho et al. 150321042* | О |  | О | О |
| 321044 | Kozushima Island | | 21-Mar-15 | *SKK Cho et al. 150321044* | О |  | О |  |
| 323003 | Izu Peninsula | | 23-Mar-15 | *SKK Cho et al. 150323003* |  |  | О |  |
| 323013 | Izu Peninsula | | 23-Mar-15 | *SKK Cho et al. 150323013* |  |  | О |  |
| 323028 | Izu Peninsula | | 23-Mar-15 | *SKK Cho et al. 150323028* |  |  | О |  |
| 323005 | Izu Peninsula | | 23-Mar-15 | *SKK Cho et al. 150323005* | О |  | О | О |
| 323007 | Izu Peninsula | | 23-Mar-15 | *SKK Cho et al. 150323007* | О |  | О | О |
| 323017 | Izu Peninsula | | 23-Mar-15 | *SKK Cho et al. 150323017* | О |  | О |  |
| 323024 | Izu Peninsula | | 23-Mar-15 | *SKK Cho et al. 150323024* | О |  | О |  |
| ***Prunus takesimensis* Nakai collected from Ulleung Island, Korea, section *Pseudocerasus* Koehne** | | | | | | | | |
| 18_001 | Cheonbu | | 21-Apr-13 | *SKK Cho et al. 130421001* | О |  |  |  |
| 18_19 | Cheonbu | | 21-Apr-13 | *SKK Cho et al. 130421019* | О |  |  |  |
| 20_37 | Naesujeon | | 22-Apr-13 | *SKK Cho et al. 130422037* | О |  |  |  |
| 2115_1 | Anpyeongjeon | | 24-Apr-14 | *SKK Cho et al. 130424001* | О |  |  |  |
| 2115_6 | Anpyeongjeon | | 24-Apr-14 | *SKK Cho et al. 130424006* | О |  |  |  |
| 21_63 | Dokdo Jeonmangdae | | 22-Apr-13 | *SKK Cho et al. 130422063* | О |  |  |  |
| 21_68 | Dokdo Jeonmangdae | | 22-Apr-13 | *SKK Cho et al. 130422068* | О |  |  |  |
| 23_80 | Bongrae Falls | | 22-Apr-13 | *SKK Cho et al. 130422080* | О |  |  |  |
| 24_101 | Taeha | | 23-Apr-13 | *SKK Cho et al. 130423101* | О |  |  |  |
| 25_116 | Nari | | 23-Apr-13 | *SKK Cho et al. 130423116* | О |  |  |  |
| 26_129 | Nari | | 23-Apr-13 | *SKK Cho et al. 130423129* | О |  |  |  |
| 27_135 | Nari | | 23-Apr-13 | *SKK Cho et al. 130423135* | О |  |  |  |
| 28_158 | Namseo-ri | | 23-Apr-13 | *SKK Cho et al. 130423158* | О |  |  |  |
| 32_197 | Sadong-ri | | 23-Apr-13 | *SKK Cho et al. 130423197* | О |  |  |  |
| 33_209 | Namseo-ri | | 24-Apr-13 | *SKK Cho et al. 130424209* | О |  |  |  |
| 514_145 | Dodong-ri | | 09-Aug-13 | *SKK Cho et al. 130809145* | О |  |  |  |
| 515_125 | Dokdo Jeonmangdae | | 09-Aug-13 | *SKK Cho et al. 130809125* | О |  |  |  |
| 515_126 | Dokdo Jeonmangdae | | 09-Aug-13 | *SKK Cho et al. 130809126* | О |  |  |  |
| 521_116 | Nari | | 10-Aug-13 | *SKK Cho et al. 130810116* | О |  |  |  |
| 521_117 | Nari | | 10-Aug-13 | *SKK Cho et al. 130810117* | О |  |  |  |
| 524_139 | Nari | | 10-Aug-13 | *SKK Cho et al. 130810139* | О |  |  |  |
| 530_129 | Cheonbu-ri | | 10-Aug-13 | *SKK Cho et al. 130810129* | О |  |  |  |
| 534_232 | Hyunpo-ri | | 10-Aug-13 | *SKK Cho et al. 130810232* | О |  |  |  |
| 534_241 | Hyunpo-ri | | 10-Aug-13 | *SKK Cho et al. 130810241* | О |  |  |  |
| 534_245 | Hyunpo-ri | | 10-Aug-13 | *SKK Cho et al. 130810245* | О |  |  |  |
| 537_244 | Bongrae Falls | | 11-Aug-13 | *SKK Cho et al. 130811244* | О |  |  |  |
| 540_292 | Seonginbong | | 11-Aug-13 | *SKK Cho et al. 130811292* | О |  |  |  |
| 540_294 | Seonginbong | | 11-Aug-13 | *SKK Cho et al. 130811294* | О |  |  |  |
| 549_169 | Namyang-ri | | 11-Aug-13 | *SKK Cho et al. 130811169* | О |  |  |  |
| 555_110 | Taeharyeong | | 11-Aug-13 | *SKK Cho et al. 130811110* | О |  |  |  |
| 555_111 | Taeharyeong | | 11-Aug-13 | *SKK Cho et al. 130811111* | О |  |  |  |
| 969_002 | Seonginbong | | 24-Apr-14 | *SKK Cho et al. 140424002* | О |  |  |  |
| 969_004 | Seonginbong | | 24-Apr-14 | *SKK Cho et al. 140424004* | О |  |  |  |
| 969_006 | Seonginbong | | 24-Apr-14 | *SKK Cho et al. 140424006* | О |  |  |  |
| 969_005 | Seonginbong | | 24-Apr-14 | *SKK Cho et al. 140424005* |  |  | О | О |
| ***Prunus apetala*(Siebold & Zucc.) Franch & Sav. collected from Japan, section *Pseudocerasus* Koehne** | | | | | | | | |
| 13011 | Tochigi, | | 2-May-13 | *SKK Cho et al. 13011* | О |  |  |  |
| 1TOHO | Tohoku Univ. Botanical Garden, Sendai | | 08-May-12 | None | О |  |  |  |
| 501014 | Tsuruoka, | | 01-May-13 | *SKK Cho et al. 130501014* | О |  |  |  |
| ***Prunus avium*(L.) L. collected from Japan, section *Eurocerasus* Koehne** | | | | | | | | |
| 13067 | Aomori, | | 2013-05-10 | *SKK Cho et al. 13067* | О |  |  |  |
| ***Prunus incisa* Thunb. collected from Japan, section *Pseudocerasus* Koehne** | | | | | | | | |
| 423036 | Tohoku Univ. Botanical Garden | | 23-Apr-13 | *SKK Cho et al. 130423036* | О |  |  |  |
| **Cloned ITS amplified accessions for the representative species, total 144 accessions** | | | | | | | | |
| ***Prunus spachiana*f.*ascendens*(Makino) Kitam. collected from Jeju Island, Korea** | | | | | | | | |
| 820 | 9 accessions | Jedong Ranch | 14-Apr-11 | *SKK Cho et al. 110414129* | О |  |  |  |
| 857 | 8 | Youngsil | 02-May-11 | *SKK Cho et al. 110502134* | О |  |  |  |
| ***Prunus yedoensis* M., wild and endemic to Jeju Island, Korea** | | | | | | | | |
| 826_1 | 8 | Bonggae Wangbeot Natural Stand | 13-Apr-11 | *SKK Cho et al. 110413106* | О |  |  |  |
| 833_2 | 8 | Kwaneumsa | 19-Apr-11 | *SKK Cho et al. 110419108* | О |  |  |  |
| 868 | 8 | Sinrye-ri, Wangbeot Natural Stand | 03-May-11 | *SKK Cho et al. 110503109* | О |  |  |  |
| 880 | 8 | Eorimok | 03-May-11 | *SKK Cho et al. 110503115* | О |  |  |  |
| **Cultivated *Prunus* ×*yedoensis* M., collected from Japan and Korea** | | | | | | | | |
| SNU_K | 10 | Seoul National University | 26-Apr-12 | *SKK Cho et al. 120426121* | О |  |  |  |
| KO3492 | 10 | Koishikawa Botanical Garden | 18-Apr-13 | None | О |  |  |  |
| KO4972 | 10 | Koishikawa Botanical Garden | 18-Apr-13 | None | О |  |  |  |
| KO4981 | 10 | Koishikawa Botanical Garden | 18-Apr-13 | None | О |  |  |  |
| KO5041 | 10 | Koishikawa Botanical Garden | 18-Apr-13 | None | О |  |  |  |
| ***Prunus sargentii* Rehder collected from Jeju Island, Korea** | | | | | | | | |
| 861 | 10 | 1100 Rest area | 02-May-11 | *SKK Cho et al. 110502201* | О |  |  |  |
| ***Prunus serrulata*var.*spontanea*(Maxim.) E.H.Wilson collected from Jeju Island, Korea** | | | | | | | | |
| 816 | 8 | Hannam-ri | 13-Apr-11 | *SKK Cho et al. 110413148* | О |  |  |  |
| ***Prunus serrulata var. quelpaertensis* (Nakai) Uyeki collected from Jeju Island, Korea** | | | | | | | | |
| 838 | 8 | Sancheondan | 19-Apr-11 | *SKK Cho et al. 110419156* | О |  |  |  |
| ***Prunus speciosa* (Koidz.) Ingram collected from Japan** | | | | | | | | |
| KO89_00277 | 9 | Koishikawa Botanical Garden | 18-Apr-13 | None | О |  |  |  |
| KO89_251 | 10 | Koishikawa Botanical Garden | 18-Apr-13 | None | О |  |  |  |
| **Cloned amplified accessions for POLA1_19 intron region, total 25 accessions** | | | | | | | | |
| ***Prunus yedoensis* M., wild and endemic to Jeju Island, Korea** | | | | | | | | |
| 5191000 | 3 | Bonggae-dong | 19-May-15 | *SKK Cho et al. 150519200* |  |  |  | О |
| 868 | 6 | Sinrye-ri, Wangbeot Natural Stand | 03-May-11 | *SKK Cho et al. 110503109* |  |  |  | О |
| **Cultivated *Prunus* ×*yedoensis* M., collected from Japan and Korea** | | | | | | | | |
| KO4972 | 8 | Koishikawa Botanical Garden | 18-Apr-13 | None |  |  |  | О |
| KO5033 | 4 | Koishikawa Botanical Garden | 18-Apr-13 | None |  |  |  | О |
| ***Prunus serrulata var. quelpaertensis* (Nakai) Uyeki collected from Jeju Island, Korea** | | | | | | | | |
| 838 | 4 | Sancheondan | 19-Apr-11 | *SKK Cho et al. 110419156* |  |  |  | О |


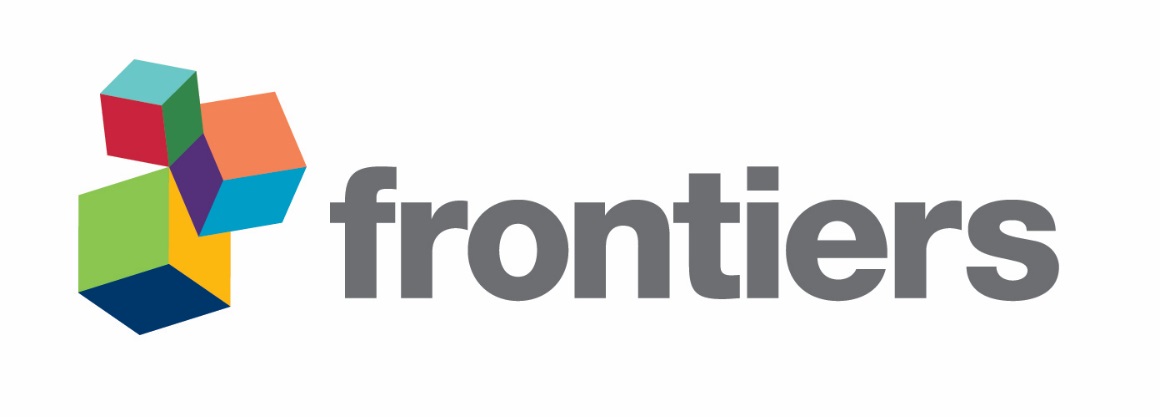

Supplement: Supplementary file 1 [file Table_1.docx]
