# Supplementary material for: Multiple Lines of Evidence for Independent Origin of Wild and Cultivated Flowering Cherry (Prunus yedoensis)
Source: Front Plant Sci. 2019 Dec 19;10:1555. doi: 10.3389/fpls.2019.01555 (PMC6930925; doi:10.3389/fpls.2019.01555)

Supplementary Material

Multiple lines of evidence for independent origin of wild and cultivated flowering cherry (*Prunus yedoensis*)

Myong-Suk Cho and Seung-Chul Kim^*^

*** Correspondence**: Seung-Chul Kim: sonchus96@skku.edu

# Supplementary Figure and Tables

## 1.2 Supplementary Tables

**Supplementary Table S2**. List of primer pairs used for amplification in this study.

| **Locus** | **Primer name** | **Primer sequences (from 5’ to 3’)** | **Reference** |
| --- | --- | --- | --- |
| **Nuclear DNA regions used for phylogenetic analyses (MP, ML, BI and SNPs) among wild *P. yedoensis* and cultivated *P.* ×*yedoensis* lineages** | | | |
| ITS1/ ITS2  intergenic spacers | ITS5 | GGAAGTAAAAGTCGTAACAAGG | White et al., 1990 |
|  | ITS4 | TCCTCCGCTTATTGATATGC |  |
|  | ITS2 | GCTGCGTTCTTCATCGATGC |  |
|  | ITS3 | GCATCGATGAAGAACGCAGC |  |
| ETS | 18S/ETS | **18S:** TGACTACTGGCAGGATCAACCAG  **ETS-ROS:** GTGAGTGGTGAATGGGTTGT | Oh and Potter, 2005 |
| POLA1  19 intron | 19ex5P/20ex3P | **19ex5P:** CTCGCTGGACGGGGTGAGATGAATG  **20ex3P:** TTGAAGATGTTCAGGTATGGGGAG | Zhang et al., 2008 |
| POLA1  20 exon | 20ex5P/21ex3P | **20ex5P:** ATAAGTTGAAGAAAATCACTGTGG  **21ex3P:** ATTTACTGGCAATCCAAGACAGAT | Zhang et al., 2008 |
| Bin (Linkage group) [8.6](http://bioinfo.bch.msu.edu/rosaceae_cos/markers?q=8:60) | [RosCOS00517](http://bioinfo.bch.msu.edu/cgi-bin/gbrowse/Rosaceae/?name=ROSC_FMLY_CSA1_517) | **forward:** CCAGAAAAGCGTCCTTCTCA  **reverse:** AATGCCGACATGGAATAGGA | Cabrera et al., 2009 |
| Bin (Linkage group) [5:08](http://bioinfo.bch.msu.edu/rosaceae_cos/markers?q=5:08) | [RosCOS01167](http://bioinfo.bch.msu.edu/cgi-bin/gbrowse/Rosaceae/?name=ROSC_FMLY_CSA1_1167) | **forward:** CCCTTCCTCTTGATTGCAGA  **reverse:** CGGATCTTTAGCTTGGCAAC | Cabrera et al., 2009 |
| Bin (Linkage group) [5:41](http://bioinfo.bch.msu.edu/rosaceae_cos/markers?q=5:41) | [RosCOS01445](http://bioinfo.bch.msu.edu/cgi-bin/gbrowse/Rosaceae/?name=ROSC_FMLY_CSA1_1445) | **forward:** TTGATCTGGCCTCAAGGAAC  **reverse:** AGATGCCATCCTCCAATACG | Cabrera et al., 2009 |
| Bin (Linkage group) [7:48](http://bioinfo.bch.msu.edu/rosaceae_cos/markers?q=7:48) | [RosCOS03628](http://bioinfo.bch.msu.edu/cgi-bin/gbrowse/Rosaceae/?name=ROSC_FMLY_CSA1_3628) | **forward:** TGGTTATTCATTCTGGATTGG  **reverse:** GCATGGCCAGGATAGTTTGT | Cabrera et al., 2009 |
| **Chloroplast noncoding DNA regions used for phylogenetic analyses (MP, ML, and BI) among wild *P. yedoensis* and cultivated *P.* ×*yedoensis* lineages** | | | |
| *psb*J-*pet*A intergenic | psbJ/petA | **psbJ:** ATAGGTACTGTARCYGGTATT  **petA:** AACARTTYGARAAGGTTCAATT | Shaw et al., 2007 |
| *pet*D-*rpo*A intergenic | petD/rpoA | **petD:** GGGCATTGGTGCAACATTAC  **rpoA:** CAGCCAAGAAGATCTTATGA | Nishizawa, 2000 |
| *ndh*F-*rpl*32 intergenic | ndhF/rpl32-R | **ndhF**: GAAAGGTATKATCCAYGMATATT  **rpL32-R:** CCAATATCCCTTYYTTTTCCAA | Shaw et al., 2007 |
| *trn*Q-*rps*16 intergenic | trnQ/rps16x1 | **trnQ^(UUG)^:** GCGTGGCCAAGYGGTAAGGC  **rpS16x1:** GTTGCTTTYTACCACATCGTTT | Shaw et al., 2007 |
| *trn*V-*ndh*C intergenic | trnV/ndhC | **trnV^(UAC)^x2:** GTCTACGGTTCGARTCCGTA  **ndhC:** TATTATTAGAAATGYCCARAAAATATCATATTC | Shaw et al., 2007 |
| *rpl*16 intron | rpL16F71/rpL16R1516 | **rpL16F71:** GCTATGCTTAGTGTGTGACTCGTTG  **rpL16R1516:** CCCTTCATTCTTCCTCTATGTTG | Shaw et al., 2005 |
| *rpl*32-*trn*L intergenic | rpl32-F/trnL | **trnL^(UAG)^:** CTGCTTCCTAAGAGCAGCGT  **rpL32-F:** CAGTTCCAAAAAAACGTACTTC | Shaw et al., 2007 |
| **Chloroplast DNA regions used for cpDNA haplotype network analysis** | | | |
| *pet*B-*pet*D intergenic | 78031F/79027R | **78031F:** AGCAAGGTATTTCTGGTCCTTT  **79027R:** ACACAGGATCATTCAAGTCAGG | Designed in this study |
| *pet*D-*rpo*A intergenic | petD/rpoA | **petD:** GGGCATTGGTGCAACATTAC  **rpoA:** CAGCCAAGAAGATCTTATGA | Nishizawa, 2000 |
| *rpl*16 intron | rpl16F/R | **rpl16F:** TTGCTTCGGTAGATATGCTCTTC  **rpl16R:** CAAAGACCCCTTTCCTTTGT | Roh et al., 2007 |
| *trn*S-*trn*G intergenic | trnS/trnG | **trnS:** AAC TCG TAC AAC GGA TTA GCA ATC  **trnG:** GAA TCG AAC CCG CAT CGT TAG | Shaw et al., 2007 |
| *ycf*1 gene | 126890F/128254R | **126890F:** CGTGTGTGCATCTCTTTGTCG  **128254R:** GGTCGATATTGACGCCTGGA | Designed in this study |

**References**

Cabrera, A., Kozik, A., Howad, W., Arus, P., Iezzoni, A. F., and van der Knaap, E. (2009). [Development and bin mapping of a Rosaceae Conserved Ortholog Set (COS) of markers](http://www.biomedcentral.com/1471-2164/10/562). *BMC genomics* 10, 562.

Nishizawa, T. (2000). Primer pairs suitable for PCR-SSCP analysis of chloroplast DNA in angiosperms. *J. Phytogeogr. Taxon*. 48, 63-66.

Oh, S. H., and Potter, D. (2005). Molecular phylogenetic systematics and biogeography of tribe Neillieae (Rosaceae) using DNA sequences of cpDNA, rDNA, and LEAFY. *Am. J. Bot.*  92, 179-192.

Roh, M. S., Cheong, E. J., Choi, I. Y., and Joung, Y. H. (2007). Characterization of wild *Prunus yedoensis* analyzed by inter-simple sequence repeat and chloroplast DNA. *Sci. Hortic.*  114, 121-128.

Shaw, J., Lickey, E. B., Beck, J. T., Farmer, S. B., Liu, W., Miller, J., Siripun, K. C., Winder, C. T., Schilling, E. E., and Small, R. L. (2005). The tortoise and hare II: relative utility of 21 noncoding chloroplast DNA sequences for phylogenetic analysis. *Am. J. Bot.*  92, 142-166.

Shaw, J., Lickey, E. B., Schilling, E. E., and Small, R. L. (2007). Comparison of whole chloroplast genome sequences to choose noncoding regions for phylogenetic studies in angiosperms, the tortoise and the hare III. *Am. J. Bot.*  94, 275-288.

White, T. J., Bruns, T., Lee S., and Taylor, J. W. (1990). Amplification and direct sequencing of fungal ribosomal RNA genes for phylogenetics. in PCR protocols, a guide to methods and applications. eds. Innis, M. A., Gelfand, D. H., Sninsky, J. J., and White, T. J. (New York: Academic Press). 315-322.

Zhang, X., Takahashi, H., Nakamura, I., and Mii, M. (2008). Molecular discrimination among taxa of *Petunia axillaris* complex and *P.* *integrifolia* complex based on PolA1 sequence analysis. *Breed. Sci.* 58, 71-75.


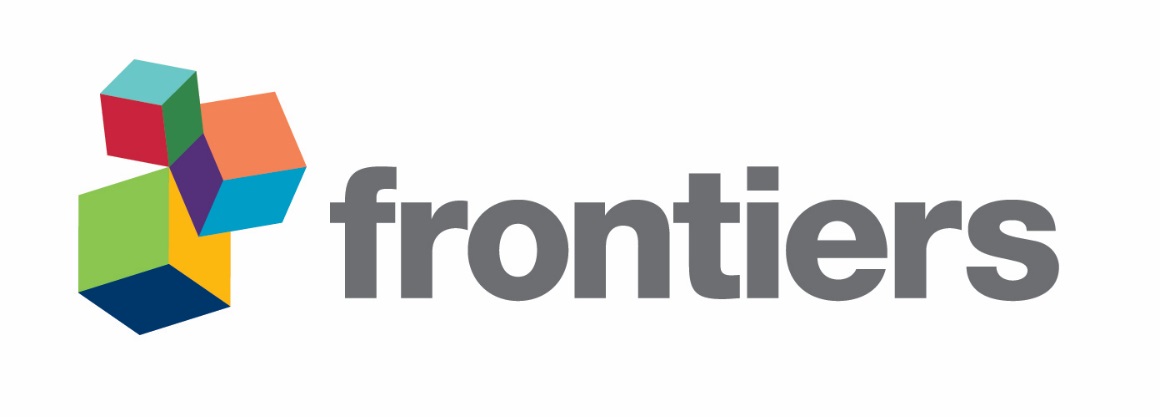

Supplement: Supplementary file 2 [file Table_2.docx]
