# Supplementary material for: Multiple Lines of Evidence for Independent Origin of Wild and Cultivated Flowering Cherry (Prunus yedoensis)
Source: Front Plant Sci. 2019 Dec 19;10:1555. doi: 10.3389/fpls.2019.01555 (PMC6930925; doi:10.3389/fpls.2019.01555)

Supplementary Material

Multiple lines of evidence for independent origin of wild and cultivated flowering cherry (*Prunus yedoensis*)

Myong-Suk Cho and Seung-Chul Kim^*^

*** Correspondence**: Seung-Chul Kim: sonchus96@skku.edu

# Supplementary Figure and Tables

## 1.2 Supplementary Tables

**Supplementary Table S3**. Twenty-six RosCOS markers and primer information used for screening eight representative species in this study.

|  | [**Name**](http://bioinfo.bch.msu.edu/rosaceae_cos/markers?c=markers.name&d=up&q=1:14) | **Forward Primer** | **Reverse Primer** | [**Bin/linkage group**](http://bioinfo.bch.msu.edu/rosaceae_cos/markers?c=bins.name&d=up&q=1:14) |
| --- | --- | --- | --- | --- |
| 1 | [RosCOS_00540](http://bioinfo.bch.msu.edu/cgi-bin/gbrowse/Rosaceae/?name=ROSC_FMLY_CSA1_540) | TTGCAAGAAGCCTGGCCTA | TTTGATAAGATCCCTAGCTTTGACA | [1:50](http://bioinfo.bch.msu.edu/rosaceae_cos/markers?q=1:50) |
| 2 | [RosCOS_01475](http://bioinfo.bch.msu.edu/cgi-bin/gbrowse/Rosaceae/?name=ROSC_FMLY_CSA1_1475) | CACTCGTGCATCTCAGCTTC | CCTGAATGGCATCAACCAG | [1:52](http://bioinfo.bch.msu.edu/rosaceae_cos/markers?q=1:52) |
| 3 | RosCOS_02072 | GCTGAAATGCATAAAGCTGGA | TGCCAGCTGCAACTGATAAC | [2:25](http://bioinfo.bch.msu.edu/rosaceae_cos/markers?q=2:25) |
| 4 | [RosCOS_01116](http://bioinfo.bch.msu.edu/cgi-bin/gbrowse/Rosaceae/?name=ROSC_FMLY_CSA1_1116) | TTGGGCATTCATGTTTGTTC | CCTTGTAGATCCATCAACGACA | [2:26](http://bioinfo.bch.msu.edu/rosaceae_cos/markers?q=2:26) |
| 5 | [RosCOS_01276](http://bioinfo.bch.msu.edu/cgi-bin/gbrowse/Rosaceae/?name=ROSC_FMLY_CSA1_1276) | GAGGGCAAATTTGATGCAGT | AGTGGAGGCCAATCCTTCTT | [2:28](http://bioinfo.bch.msu.edu/rosaceae_cos/markers?q=2:28) |
| 6 | [RosCOS_01480](http://bioinfo.bch.msu.edu/cgi-bin/gbrowse/Rosaceae/?name=ROSC_FMLY_CSA1_1480) | TTTGAGAAACAAACCGATTGAA | TCTGTGCATCCCAGTACGAG | [3:22](http://bioinfo.bch.msu.edu/rosaceae_cos/markers?q=3:22) |
| 7 | [RosCOS_01134](http://bioinfo.bch.msu.edu/cgi-bin/gbrowse/Rosaceae/?name=ROSC_FMLY_CSA1_1134) | CTGGTGACCTAGGCCCATTA | CCGAGCATGCATAAAAGGAT | [3:37](http://bioinfo.bch.msu.edu/rosaceae_cos/markers?q=3:37) |
| 8 | [RosCOS_03763](http://bioinfo.bch.msu.edu/cgi-bin/gbrowse/Rosaceae/?name=ROSC_FMLY_CSA1_3763) | GCGTGAGAGGATTCTGGCTA | CCTTCTTTTGGAGGACCGTA | [3:49](http://bioinfo.bch.msu.edu/rosaceae_cos/markers?q=3:49) |
| 9 | [RosCOS_01163](http://bioinfo.bch.msu.edu/cgi-bin/gbrowse/Rosaceae/?name=ROSC_FMLY_CSA1_1163) | TCTTTGGAATTGCCTCTGCT | CATCCACATGCTGACGAAAG | [4:18](http://bioinfo.bch.msu.edu/rosaceae_cos/markers?q=4:18) |
| 10 | [RosCOS_03739](http://bioinfo.bch.msu.edu/cgi-bin/gbrowse/Rosaceae/?name=ROSC_FMLY_CSA1_3739) | GGCAGAAGACTGCAGGAAGTA | GCATTTGGGACAAAGAGACC | [4:27](http://bioinfo.bch.msu.edu/rosaceae_cos/markers?q=4:27) |
| 11 | [RosCOS_03576](http://bioinfo.bch.msu.edu/cgi-bin/gbrowse/Rosaceae/?name=ROSC_FMLY_CSA1_3576) | AATGTAGTTGCTTGGCATGG | GGCCCATAAATTCGCTCATA | [4:46](http://bioinfo.bch.msu.edu/rosaceae_cos/markers?q=4:46) |
| 12 | [RosCOS_01339](http://bioinfo.bch.msu.edu/cgi-bin/gbrowse/Rosaceae/?name=ROSC_FMLY_CSA1_1339) | ATGGGGAACTGTTGGATTGA | GCTCAAGGTCCTTTGATTGAA | 4.63 |
| 13 | [RosCOS_01167](http://bioinfo.bch.msu.edu/cgi-bin/gbrowse/Rosaceae/?name=ROSC_FMLY_CSA1_1167) | CCCTTCCTCTTGATTGCAGA | CGGATCTTTAGCTTGGCAAC | [5:08](http://bioinfo.bch.msu.edu/rosaceae_cos/markers?q=5:08) |
| 14 | [RosCOS_03799](http://bioinfo.bch.msu.edu/cgi-bin/gbrowse/Rosaceae/?name=ROSC_FMLY_CSA1_3799) | ATCGCAACATCCTTGACAAA | TGGTTCTGATCCACCTCACA | [5:21](http://bioinfo.bch.msu.edu/rosaceae_cos/markers?q=5:21) |
| 15 | [RosCOS_01445](http://bioinfo.bch.msu.edu/cgi-bin/gbrowse/Rosaceae/?name=ROSC_FMLY_CSA1_1445) | TTGATCTGGCCTCAAGGAAC | AGATGCCATCCTCCAATACG | [5:41](http://bioinfo.bch.msu.edu/rosaceae_cos/markers?q=5:41) |
| 16 | [RosCOS_00643](http://bioinfo.bch.msu.edu/cgi-bin/gbrowse/Rosaceae/?name=ROSC_FMLY_CSA1_643) | GCGTGGCAGCTATTAACACA | TCACCAAACTCTGATTTCATCATT | [5:49](http://bioinfo.bch.msu.edu/rosaceae_cos/markers?q=5:49) |
| 17 | [RosCOS_01240](http://bioinfo.bch.msu.edu/cgi-bin/gbrowse/Rosaceae/?name=ROSC_FMLY_CSA1_1240) | CGGTAAACCCAGGAGCTTTT | ATCTTGTTGCCGTCGAAGTT | [6:25](http://bioinfo.bch.msu.edu/rosaceae_cos/markers?q=6:25) |
| 18 | [RosCOS_01493](http://bioinfo.bch.msu.edu/cgi-bin/gbrowse/Rosaceae/?name=ROSC_FMLY_CSA1_1493) | GGGGAACTAAATCACAGCTCA | GAAGCAAGCCCAACCAAG | 6.84 |
| 19 | [RosCOS_02599](http://bioinfo.bch.msu.edu/cgi-bin/gbrowse/Rosaceae/?name=ROSC_FMLY_CSA1_2599) | GTTCGCTCCTGTTGAGGAAG | CTGACGGCCAGACTCTAGGA | [7:25](http://bioinfo.bch.msu.edu/rosaceae_cos/markers?q=7:25) |
| 20 | [RosCOS_03628](http://bioinfo.bch.msu.edu/cgi-bin/gbrowse/Rosaceae/?name=ROSC_FMLY_CSA1_3628) | TGGTTATTCATTCTGGATTGG | GCATGGCCAGGATAGTTTGT | [7:48](http://bioinfo.bch.msu.edu/rosaceae_cos/markers?q=7:48) |
| 21 | [RosCOS_01250](http://bioinfo.bch.msu.edu/cgi-bin/gbrowse/Rosaceae/?name=ROSC_FMLY_CSA1_1250) | TCATGGGGATTGGTAACACC | CCTGGTCAAGTTCCACATTG | [7:56](http://bioinfo.bch.msu.edu/rosaceae_cos/markers?q=7:56) |
| 22 | [RosCOS_01302](http://bioinfo.bch.msu.edu/cgi-bin/gbrowse/Rosaceae/?name=ROSC_FMLY_CSA1_1302) | TGCCTCCCAAATCGTAACTC | TGGGTCTCTTTCCATCCTTG | [7:56](http://bioinfo.bch.msu.edu/rosaceae_cos/markers?q=7:56) |
| 23 | [RosCOS_01200](http://bioinfo.bch.msu.edu/cgi-bin/gbrowse/Rosaceae/?name=ROSC_FMLY_CSA1_1200) | AAGAAGTGGGTGATGGCAAC | CATGCCAATCAAAGACATGG | [8:41](http://bioinfo.bch.msu.edu/rosaceae_cos/markers?q=8:41) |
| 24 | [RosCOS_03732](http://bioinfo.bch.msu.edu/cgi-bin/gbrowse/Rosaceae/?name=ROSC_FMLY_CSA1_3732) | ACAGCTGCGATCCAAGTAGC | CCGAAATCAACAATATCAAGAGC | [8:41](http://bioinfo.bch.msu.edu/rosaceae_cos/markers?q=8:41) |
| 25 | [RosCOS_01338](http://bioinfo.bch.msu.edu/cgi-bin/gbrowse/Rosaceae/?name=ROSC_FMLY_CSA1_1338) | GACGCCAAAGACATGAGGAT | CCAGAAGCAAACCCATTGTT | [8:41](http://bioinfo.bch.msu.edu/rosaceae_cos/markers?q=8:41) |
| 26 | [RosCOS_00517](http://bioinfo.bch.msu.edu/cgi-bin/gbrowse/Rosaceae/?name=ROSC_FMLY_CSA1_517) | CCAGAAAAGCGTCCTTCTCA | AATGCCGACATGGAATAGGA | [8.6](http://bioinfo.bch.msu.edu/rosaceae_cos/markers?q=8:60) |


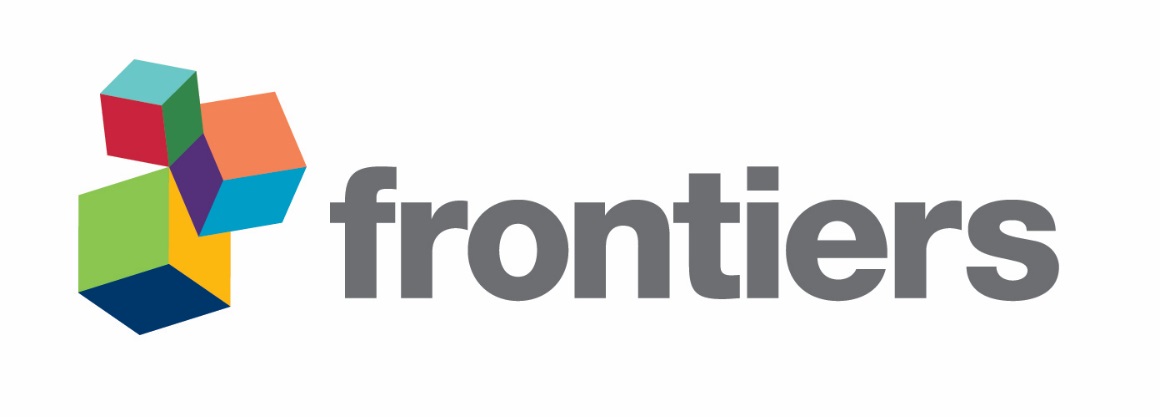

Supplement: Supplementary file 3 [file Table_3.docx]
