# Supplementary material for: Multiple Lines of Evidence for Independent Origin of Wild and Cultivated Flowering Cherry (Prunus yedoensis)
Source: Front Plant Sci. 2019 Dec 19;10:1555. doi: 10.3389/fpls.2019.01555 (PMC6930925; doi:10.3389/fpls.2019.01555)

Supplementary Material

Multiple lines of evidence for independent origin of wild and cultivated flowering cherry (*Prunus yedoensis*)

Myong-Suk Cho and Seung-Chul Kim^*^

*** Correspondence**: Seung-Chul Kim: sonchus96@skku.edu

# Supplementary Figure and Tables

## 1.2 Supplementary Tables

**Supplementary Table S4**. Additive polymorphic sites (APS) from the concatenated ITS and ETS sequences among wild and cultivated *P. yedoensis* and their putative parental lineages. A total of 156 out of 194 individuals were used in the analyses, excluding outgroup species and *P. takesimensis* from Ulleung Island. Nucleotides that were less frequent or rare among individuals are indicated by asterisks (*).

| *Prunus* Species | Sample  numbers | Informative ITS nucleotide site | | | | | | | | | | Informative ETS  nucleotide site | | |
| --- | --- | --- | --- | --- | --- | --- | --- | --- | --- | --- | --- | --- | --- | --- |
|  |  | 41 | 44 | 63 | 96 | 164 | 185 | 191 | 438 | 567 | 573 | 687 | 759 | 913 |
| *P. spachiana* f. *ascendens* Jeju | 25 pcs | C | A | T/Y* | G | T | T/Y* | C | G/K* | A | A | C/Y* | A/R* | A/R* |
| *P. spachiana* f. *ascendens* Japan | 12 | C | A | T | G | T | T | C | G | A | A | C | A | A |
| **Wild *P. yedoensis*, Jeju** | **17** | **M** | **M/C*** | **Y/C*** | **R/G*/A*** | **Y** | **Y** | **C/M*** | **K** | **R/A*** | **M/A*** | **Y/T*** | **R/G*** | **R/G*** |
| **Cultivated *P.* ×*yedoensis*, Japan** | **12** | **M/C*** | **M** | **Y** | **R/G*** | **Y** | **Y** | **M** | **K** | **R** | **M** | **Y** | **R** | **R** |
| *P. sargentii* Jeju | 9 | A | C | C | A/R* | C | C | M/A*/C* | T | G | C | T | G | G |
| *P. sargentii* Korean Penunsula | 14 | A | C | C | R/A* | C | C | M/A* | T | G | C | T | G | G |
| *P. sargentii* Russia | 5 | A | C | C | R/A* | C | C | M/C* | T | G | C | T | G | G |
| *P. sargentii* Japan | 8 | A | C | C | A/R* | C | C | M/A* | T | G | C | T | G | G |
| *P. serrulata* var*.spontanea* Jeju | 5 | A/M* | C | C | R/A* | C | C | A/M/C* | T | G | C | T | G | G |
| *P. serrulata* var*.spontanea* Japan | 5 | A | C | C | R/A* | C | C | M | T | G | C | T | G | G |
| *P. serrulata* var. *quelpaertensis* Jeju | 6 | A/C* | C | C | R | C/Y* | C/Y* | M/C* | T/K* | G/R* | C | T/Y* | G/R* | G |
| *P. serrulata* var. *pubescens* Korean Peninsula | 3 | A | C | C | R/A* | C | C | C | T | G | C | T | G | G |
| *P. serrulata* var. *pubescens* Japan | 1 | A | C | C | A | C | C | M | T | G | C | T | G | G |
| *P. yedoensis* var. *angustipetala* Jeju | 1 | A | C | C | G | C | C | M | T | G | C | T | G | G |
| *P. longistylus* Jeju | 1 | M | C | C | G | C | C | M | T | G | C | T | G | G |
| *P. hallasanensis* Jeju | 2 | A | C | C | A | C | C | A | T | G | C | T | G | G |
| *P. speciosa* Jeju | 3 | A | C | C | A/R* | C | C | A | T | G | C | T | G | G |
| *P. speciosa* Japan | 20 | A | C | C | R/G* | C | C | A | T | G | C | T | G | G |
| *P. maximowiczii* Jeju | 3 | A | C | C | G | C | C | C | T | G | C | T | G | G |
| *P. apetala* Japan | 2 | A | C | C | R | C | C | M | T | G | C | T | G | G |
| *P. avium* Japan | 1 | A | C | C | A | C | C | C | T | G | C | T | G | G |
| *P. incisa* Japan | 1 | A | C | C | A | C | C | A | T | G | C | T | G | G |


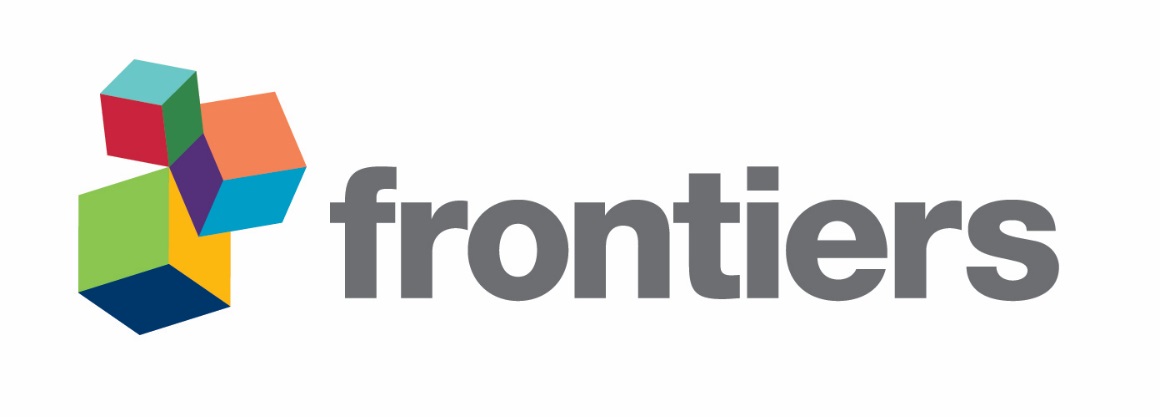

Supplement: Supplementary file 4 [file Table_4.docx]
