# Supplementary material for: Multiple Lines of Evidence for Independent Origin of Wild and Cultivated Flowering Cherry (Prunus yedoensis)
Source: Front Plant Sci. 2019 Dec 19;10:1555. doi: 10.3389/fpls.2019.01555 (PMC6930925; doi:10.3389/fpls.2019.01555)

Supplementary Material

Multiple lines of evidence for independent origin of wild and cultivated flowering cherry (*Prunus yedoensis*)

Myong-Suk Cho and Seung-Chul Kim^*^

*** Correspondence**: Seung-Chul Kim: sonchus96@skku.edu

# Supplementary Figure and Tables

## 1.2 Supplementary Tables

**Supplementary Table S5**. Single nucleotide polymorphisms (SNPs) in four RosCOS data set showing additive polymorphisms (APS) in the sequences of wild *P. yedoensis*, cultivated *P.* × *yedoensis*, and their parental lineages. Different paternal contributions between wild and cultivated *P. yedoensis* lineages are reflected in species-specific sites marked in bold type.

| Lineage | RosCOS marker | 517 | | | | | | | | | | 1167 | |
| --- | --- | --- | --- | --- | --- | --- | --- | --- | --- | --- | --- | --- | --- |
|  | Site No. | 80 | 133 | 144 | 188 | 229 | 244 | 248 | 278 | 295 | 308 | 449 | 467 |
| Korean lineage | *P. spachiana*  f. *ascendens* Jeju Island 5 samples | C | T | T | T | G | T | A | G | T | A | C | A |
|  | **wild *P. yedoensis* Jeju Island 11 samples** | **C** | **T** | **T** | **K** | **R/G*** | **T/Y*** | **A/R*** | **R** | **T/W*** | **A** | **M** | **R** |
|  | *P. serrulata* var. *quelpaertensis*  Jeju Island 8 samples | C/Y* | T/W* | T/W* | G/K* | R/A*/G* | T/Y* | A/R* | A/R* | T/W* | A/R* | M/A/C* | G/R* |
|  | *P. sargentii* Jeju 11 samples | C | T/W* | T/W* | G | R/A*/G* | T/Y* | A/R* | A | T/W*/A* | A | A/M* | G |
|  | *P. serrulata* v. *spontaneae*  Jeju Island 6 samples | C/Y* | T/W* | T/W* | G/K*R* | A/G*/R* | Y/T* | A/R* | A/R* | W/T* | A/R* | A/M/C* | G/A*/R* |
|  | *P. serrulata* v. *pubescens* 1 sample | C | T | T | G | G | C | G | A | A | A | M | G |
|  | *P. takesimensis*  Ulleung Island 1 sample | C | T | T | G | R | T | A | A | T | A | M | R |
| Japanese lineage | *P. spachiana* f. *ascendens*  6 samples | C | T/W* | T/W* | T/K* | G | T/Y* | A | G/R* | T/W* | A/R* | C | A |
|  | **cultivated *P. xyedoensis* 5 samples** | **Y** | **W** | **W** | **K** | **G** | **Y** | **A** | **R** | **W** | **R** | **C/M*** | **A/R*** |
|  | **wild *P. yedoensis* 833_1**  **(Kwaneum temple, Jeju Island)** | **Y** | **W** | **W** | **K** | **G** | **Y** | **A** | **R** | **W** | **R** | **C** | **A** |
|  | **wild *P. yedoensis* 408003**  **(Kwaneum temple, Jeju Island)** | **Y** | **W** | **W** | **K** | **G** | **Y** | **A** | **R** | **W** | **R** | **C** | **A** |
|  | *P. speciosa* 31 samples | T/Y/C* | W/A/T* | A/W*/T* | G | G/R* | C/Y/T* | A | A | A/W/T* | G/R/A* | C/M*/A* | A/R*/G* |

| Lineage | RosCOS marker | 517 | | | | | | | | | | 1167 | |
| --- | --- | --- | --- | --- | --- | --- | --- | --- | --- | --- | --- | --- | --- |
|  | Site No. | 80 | 133 | 144 | 188 | 229 | 244 | 248 | 278 | 295 | 308 | 449 | 467 |
| Korean lineage | *P. spachiana*  f. *ascendens* Jeju Island 5 samples | C | T | T | T | G | T | A | G | T | A | C | A |
|  | **wild *P. yedoensis* Jeju Island 11 samples** | **C** | **T** | **T** | **K** | **R/G*** | **T/Y*** | **A/R*** | **R** | **T/W*** | **A** | **M** | **R** |
|  | *P. serrulata* var. *quelpaertensis*  Jeju Island 8 samples | C/Y* | T/W* | T/W* | G/K* | R/A*/G* | T/Y* | A/R* | A/R* | T/W* | A/R* | M/A/C* | G/R* |
|  | *P. sargentii* Jeju 11 samples | C | T/W* | T/W* | G | R/A*/G* | T/Y* | A/R* | A | T/W*/A* | A | A/M* | G |
|  | *P. serrulata* v. *spontaneae*  Jeju Island 6 samples | C/Y* | T/W* | T/W* | G/K*R* | A/G*/R* | Y/T* | A/R* | A/R* | W/T* | A/R* | A/M/C* | G/A*/R* |
|  | *P. serrulata* v. *pubescens* 1 sample | C | T | T | G | G | C | G | A | A | A | M | G |
|  | *P. takesimensis* Ulleung Island 1 sample | C | T | T | G | R | T | A | A | T | A | M | R |
| Japanese lineage | *P. spachiana* f. *ascendens*  6 samples | C | T/W* | T/W* | T/K* | G | T/Y* | A | G/R* | T/W* | A/R* | C | A |
|  | **cultivated *P. xyedoensis* 5 samples** | **Y** | **W** | **W** | **K** | **G** | **Y** | **A** | **R** | **W** | **R** | **C/M*** | **A/R*** |
|  | **wild *P. yedoensis* 833_1**  **(Kwaneum temple, Jeju Island)** | **Y** | **W** | **W** | **K** | **G** | **Y** | **A** | **R** | **W** | **R** | **C** | **A** |
|  | **wild *P. yedoensis* 408003**  **(Kwaneum temple, Jeju Island)** | **Y** | **W** | **W** | **K** | **G** | **Y** | **A** | **R** | **W** | **R** | **C** | **A** |
|  | *P. speciosa* 31 samples | T/Y/C* | W/A/T* | A/W*/T* | G | G/R* | C/Y/T* | A | A | A/W/T* | G/R/A* | C/M*/A* | A/R*/G* |


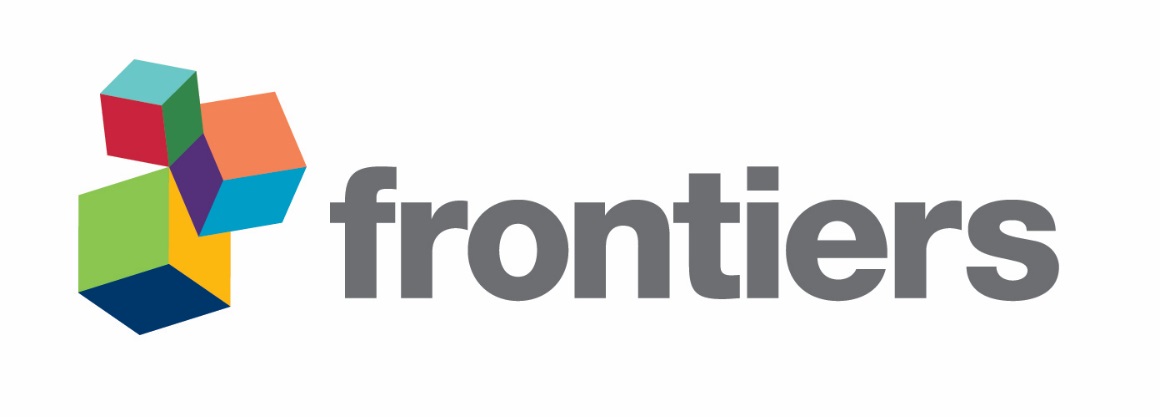

Supplement: Supplementary file 5 [file Table_5.docx]
