# Supplementary material for: Multiple Lines of Evidence for Independent Origin of Wild and Cultivated Flowering Cherry (Prunus yedoensis)
Source: Front Plant Sci. 2019 Dec 19;10:1555. doi: 10.3389/fpls.2019.01555 (PMC6930925; doi:10.3389/fpls.2019.01555)

Supplementary Material

Multiple lines of evidence for independent origin of wild and cultivated flowering cherry (*Prunus yedoensis*)

Myong-Suk Cho and Seung-Chul Kim^*^

*** Correspondence**: Seung-Chul Kim: sonchus96@skku.edu

# Supplementary Figure and Tables

## 1.2 Supplementary Tables

**Supplementary Table S6**. Single nucleotide polymorphisms (SNPs) in the *PolA1* PI19 data set. Different paternal contributions between wild and cultivated *P. yedoensis* lineages are reflected in species-specific sites marked in bold based on majority sequences found.

| Lineage | Site number | 1 | 61 | 72 | 74 | 75-76 | 81 | 139 | 166 | 169 | 184 | 209 | 212 | 216 |
| --- | --- | --- | --- | --- | --- | --- | --- | --- | --- | --- | --- | --- | --- | --- |
| Nakamura et al., 2015, data | LC010416*_P. pendula*  Cultivar: Komatsu otome | N | A | T | T | N | T | A | A | C | A | C | T | G |
|  | LC010415*_P. pendula* (K)  Cultivar: Komatsu otome | N | A | T | T | N | T | A | A | C | A | C | T | G |
|  | **LC010413*_P.yedoensis* (K)** | **N** | **A** | **T** | **T** | **N** | **T** | **A** | **A** | **C** | **A** | **C** | **T** | **G** |
|  | **LC010414*_P.yedoensis* (O)** | **N** | **T** | **C** | **C** | **N** | **A** | **T** | **G** | **T** | **G** | **A** | **C** | **A** |
|  | LC010396*_P. speciosa* | N | T | C | C | N | A | T | G | T | G | A | C | A |
|  | LC010397*_P. speciosa* | N | T | C | C | N | A | T | G | T | G | A | C | A |
| Korean lineage in this study | *P. spachiana* f. *ascendens* Jeju 5 samples | G | A | T | T | TT | T | A | A | C | A | C | T | G |
|  | **wild *P. yedoensis***  **10 samples + 9 clones** | **G** | **A** | **Y** | **Y** | **--/TT＊** | **W/T＊** | **A** | **R** | **Y** | **R** | **C** | **Y** | **R** |
|  | *P. serrulata* v. *quelpaertensis*  1 sample + 4 clones | G | A | C | C | -- | A | A | G | T | G | C | C | A |
|  | *P. serrulata* v*. spontaneae*  3 samples | G | A | C | C | -- | T/A＊ | A | G | T | R/G＊ | C | C | A |
|  | *P. sargentii* 2 samples | G | A | C | C/Y | -- | A/T | A | G | T | G/R | C | C | A |
|  | *P. serrulata* v*. pubescens*  1 sample | G | A | C | C | -- | A | A | G | T | R | C | C | A |
|  | *P. takesimensis* 1 sample | G | A | C | C | -- | A | A | G | T | G | C | C | A |
| Japanese lineage in this study | *P. spachiana* f. *ascendens* Japan 5 samples | G | A | T | T | TT | T | A | A | C | A | C | T | G |
|  | **cultivated *P.* ×*yedoensis***  **5 samples + 12 clones** | **R** | **W** | **Y** | **Y** | **--/TT＊** | **W** | **W** | **R** | **Y** | **R** | **R** | **Y** | **R** |
|  | **wild *P. yedoensis* 833_1**  **(Kwaneum temple, Jeju Island)** | **N** | **N** | **N** | **Y** | **W** | **T** | **N** | **N** | **N** | **N** | **N** | **N** | **N** |
|  | **wild *P. yedoensis* 408003**  **(Kwaneum temple, Jeju Island)** | **R** | **W** | **N** | **Y** | **W** | **T** | **N** | **N** | **N** | **N** | **N** | **N** | **R** |
|  | *P. speciosa* 6 samples | A | T/W＊ | C | C | -- | A | T/W＊ | G | T | G | A | C | A |

| Lineage | Site number | 238 | 336 | 343 | 347 | 387-386 | 430 | 454 | 492 | 495 | 515 | 530 | 538 |
| --- | --- | --- | --- | --- | --- | --- | --- | --- | --- | --- | --- | --- | --- |
| Nakamura et al., 2015, data | LC010416*_P. pendula*  Cultivar: Komatsu otome | A | T | C | T | -- | C | A | G | C | A | C | G |
|  | LC010415*_P. pendula* (K)  Cultivar: Komatsu otome | A | T | C | T | -- | A | A | G | C | A | C | G |
|  | **LC010413*_P.yedoensis* (K)** | **A** | **T** | **C** | **T** | **--** | **A** | **A** | **G** | **C** | **A** | **C** | **G** |
|  | **LC010414*_P.yedoensis* (O)** | **C** | **C** | **T** | **G** | **AA** | **C** | **T** | **G** | **A** | **G** | **T** | **A** |
|  | LC010396*_P. speciosa* | C | C | T | G | AA | C | T | G | A | G | T | A |
|  | LC010397*_P. speciosa* | C | C | T | G | AA | C | T | G | A | G | T | A |
| Korean lineage in this study | *P. spachiana* f. *ascendens* Jeju 5 samples | A | T | C | T | - | C | A | G | C | A | C | G |
|  | **wild *P. yedoensis***  **10 samples + 9 clones** | **M** | **Y** | **Y** | **K** | **--/AA** | **C** | **W** | **G/R＊** | **M/C＊** | **R** | **Y** | **R** |
|  | *P. serrulata* v. *quelpaertensis*  1 sample + 4 clones | C | C | T | G | AA | C | T | G | A | G | T | A |
|  | *P. serrulata* v*. spontaneae*  3 samples | C | C | T | G | AA | C | T | G/R＊ | C/M/A | G | T | A |
|  | *P. sargentii* 2 samples | C | C | T | G | AA | C | T | R | C/M | G | T | A |
|  | *P. serrulata* v*. pubescens*  1 sample | C | C | T | G | AA | C | T | G | A | G | T | A |
|  | *P. takesimensis* 1 sample | C | C | T | G | AA | C | T | G | A | G | T | A |
| Japanese lineage in this study | *P. spachiana* f. *ascendens* Japan 5 samples | A | T | C | T | - | C/M/A＊ | A | G | C | A | C | G |
|  | **cultivated *P.* ×*yedoensis***  **5 samples + 12 clones** | **M** | **Y** | **Y** | **K** | **--/AA** | **M** | **W** | **G** | **M** | **R** | **Y** | **R** |
|  | **wild *P. yedoensis* 833_1**  **(Kwaneum temple, Jeju Island)** | **N** | **N** | **N** | **N** | **N** | **M** | **W** | **G** | **M** | **R** | **Y** | **R** |
|  | **wild *P. yedoensis* 408003**  **(Kwaneum temple, Jeju Island)** | **N** | **N** | **N** | **N** | **N** | **M** | **W** | **G** | **M** | **R** | **Y** | **R** |
|  | *P. speciosa* 6 samples | C | C | T | G | AA | C | T | G | A | G | T | A |


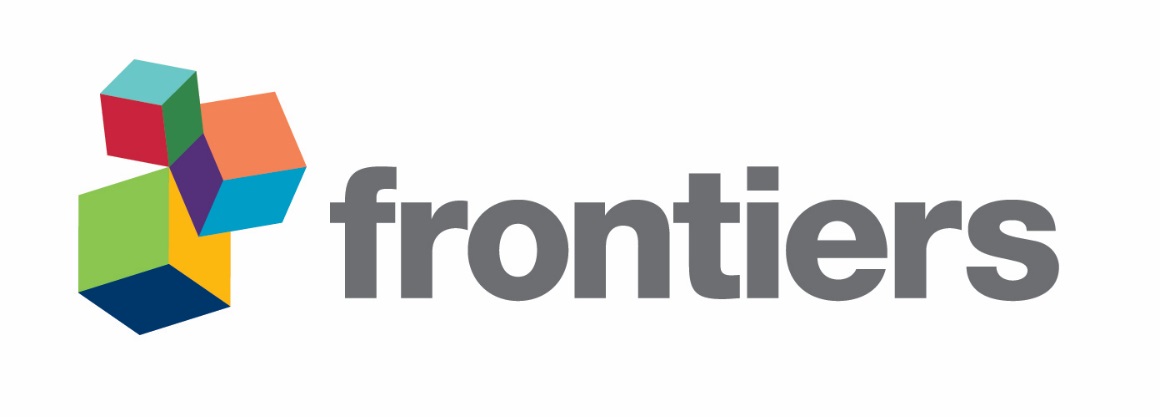

Supplement: Supplementary file 6 [file Table_6.docx]
