# Supplementary material for: Multiple Lines of Evidence for Independent Origin of Wild and Cultivated Flowering Cherry (Prunus yedoensis)
Source: Front Plant Sci. 2019 Dec 19;10:1555. doi: 10.3389/fpls.2019.01555 (PMC6930925; doi:10.3389/fpls.2019.01555)

Supplementary Material

Multiple lines of evidence for independent origin of wild and cultivated flowering cherry (*Prunus yedoensis*)

Myong-Suk Cho and Seung-Chul Kim^*^

*** Correspondence**: Seung-Chul Kim: sonchus96@skku.edu

# Supplementary Figure and Tables

## 1.2 Supplementary Tables

**Supplementary Table S7**. Single nucleotide polymorphisms (SNPs) in the *PolA1* PE20 data set. Different paternal contributions between wild and cultivated *P. yedoensis* lineages are reflected on species-specific sites marked in bold based on majority sequences found.

| Lineage | Site number | 121 | 463 | 471 | 513 | 534-557 | 676 | 705 | 712 |
| --- | --- | --- | --- | --- | --- | --- | --- | --- | --- |
| Nakamura et al., 2015, data | LC010564*_P. pendula*  Cultivar: Komatsu otome | A | A | T | C | - | A | T | C |
|  | **LC010562*_P.yedoensis* (K)** | **A** | **A** | **T** | **C** | **-** | **A** | **T** | **C** |
|  | **LC010563*_P.yedoensis* (O)** | **G** | **A** | **T** | **T** | **TTTAGAAGAAGCTGGTGGAAGTCC** | **A** | **C** | **T** |
|  | LC010553*_P. speciosa* | G | A | T | T | TTTAGAAGAAGCTGGTGGAAGTCC | A | C | T |
|  | LC010555*_P. speciosa* | G | A | T | T | TTTAGAAGAAGCTGGTGGAAGTCC | A | C | T |
| Korean lineage in this study | *P. spachiana* f. *ascendens* Jeju 5 samples | A | A | T | C | - | A | T | C |
|  | **wild *P. yedoensis* 10 samples** | **R/A＊** | **A/W＊** | **T/Y＊** | **Y** | **-/N** | **A/C＊/M＊** | **T/Y＊** | **C** |
|  | *P. serrulata* v. *quelpaertensis*  1 sample | G | A | T | T | TTTAGAAGAAGCTGGTGGAAGTCC | A | C | T |
|  | *P. serrulata* v*. spontaneae*  3 samples | A/G/R | A | T | T | -/TTTAGAAGAAGCTGGTGGAAGTCC＊ | A | T/C | C/T |
|  | *P. sargentii* 2 samples | A/R | W | Y | T | - | M | T/Y | C |
|  | *P. serrulata* v*. pubescens*  1 sample | G | A | T | T | - | A | C | C |
|  | *P. takesimensis* 1 sample | G | A | T | T | _ | A | C | T |
| Japanese lineage in this study | *P. spachiana* f. *ascendens* Japan 6 samples | A | A | T | C | - | A | T | C |
|  | **cultivated *P.* ×*yedoensis***  **5 samples** | **R** | **A** | **T** | **Y** | **-/N** | **N** | **N** | **N** |
|  | *P. speciosa* 6 samples | G | A | T | T | TTTAGAAGAAGCTGGTGGAAGTCC | A | C | T |


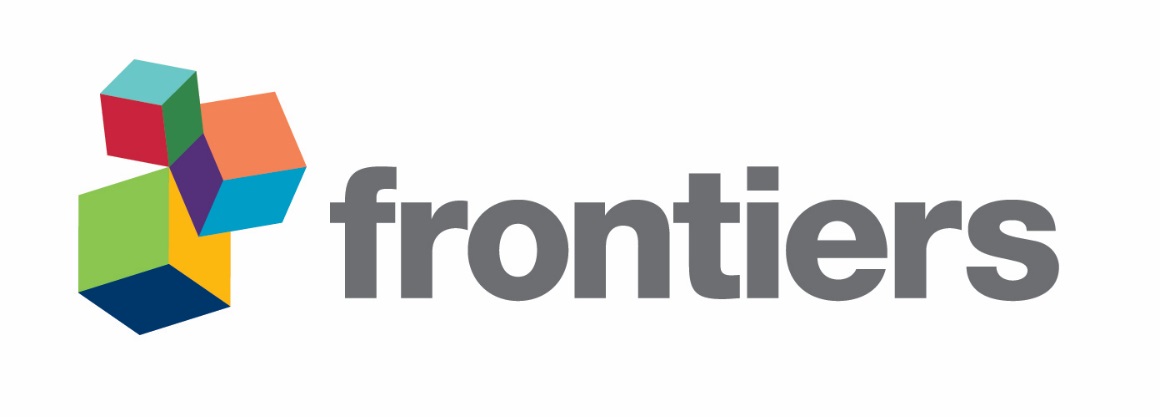

Supplement: Supplementary file 7 [file Table_7.docx]
